# Supplementary material for: The GTPase Domain of MX2 Interacts with the HIV-1 Capsid, Enabling Its Short Isoform to Moderate Antiviral Restriction
Source: Cell Rep. 2019 Nov 12;29(7):1923–1933.e3. doi: 10.1016/j.celrep.2019.10.009 (PMC7391006; doi:10.1016/j.celrep.2019.10.009)
Supplement: Document S1. Figures S1–S7 [file mmc1.pdf]

Cell Reports, Volume *29*

Supplemental Information

The GTPase Domain of MX2 Interacts with the HIV-1 Capsid, Enabling Its Short Isoform to Moderate Antiviral Restriction

Gilberto Betancor, Matthew D.J. Dicks, Jose M. Jimenez-Guardeño, Nabil H. Ali, Luis Apolonia, and Michael H. Malim

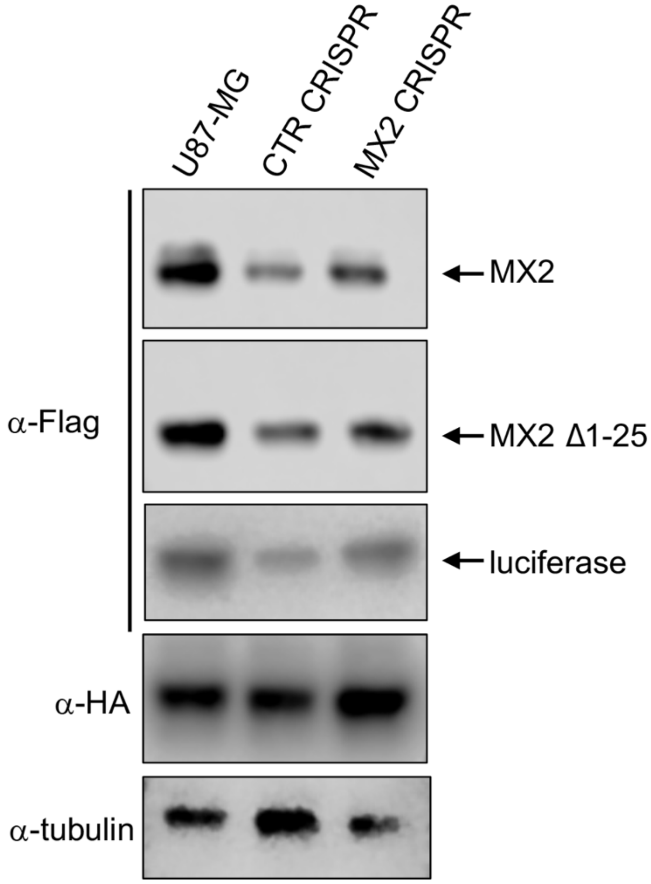

Figure S1. Protein levels in U87-MG parental cells and CTR CRISPR and MX2 CRISPR derived cell lines (related to Figure 3A-C).

Protein expression in cells transduced with Flag-tagged MX2 (resistant to CRISPR guide), MX2 Δ1-25, or Luc or HA-tagged MX2 Δ1-25 M574D was analyzed by immunoblot, with α-tubulin included as the loading control.

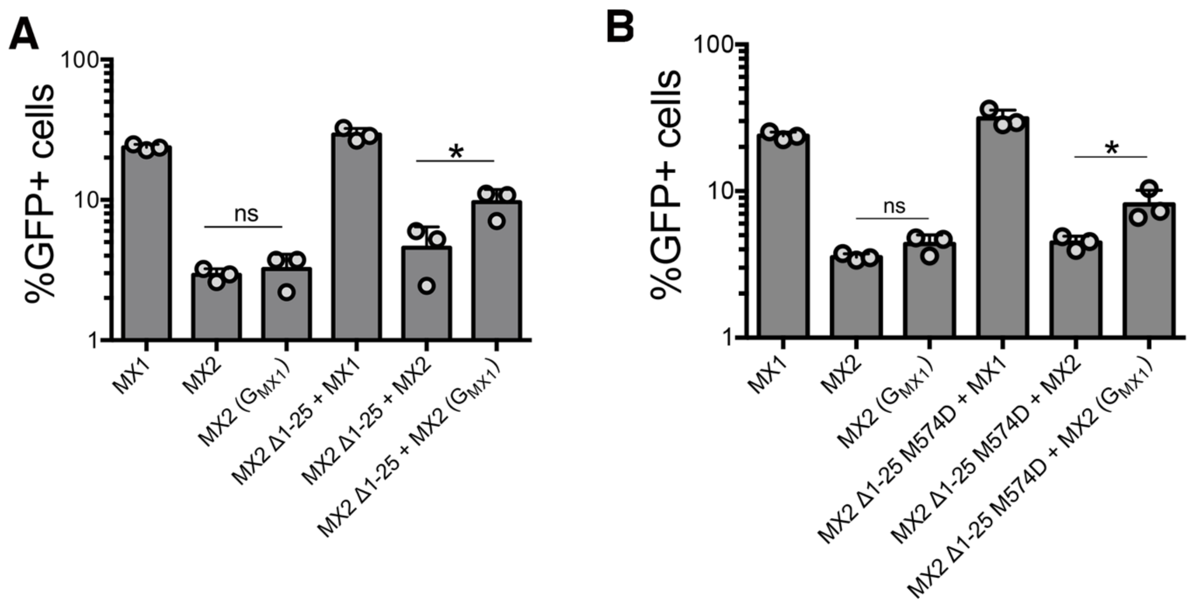

Figure S2. Wild type MX2 inhibits HIV-1 more potently than MX2 (G_{MX1}) (related to Figure 4B and C).

A U87-MG CD4/CXCR4 cells were transduced with EasiLV expressing MX2 Δ1-25 together with either MX1-, MX2- or MX2 (G_{MX1})-expressing lentiviral vectors carrying puromycinR. Two days later, transduced cells were selected with 1 μg/ml puromycin for 48 h and induced with doxycycline. Cells were then infected with an HIV-1-based lentiviral vector expressing GFP, and the percentage of GFP-expressing cells was evaluated by flow cytometry at 48 h (n = 3, mean ± SD; **p*-value < 0.05; (ns) non-significant; unpaired t-test).

B Same as A, but expressing monomeric mutant MX2 Δ1-25 M574D instead of MX2 Δ1-25 (n = 3, mean ± SD; **p*-value < 0.05; (ns) non-significant; unpaired t-test).

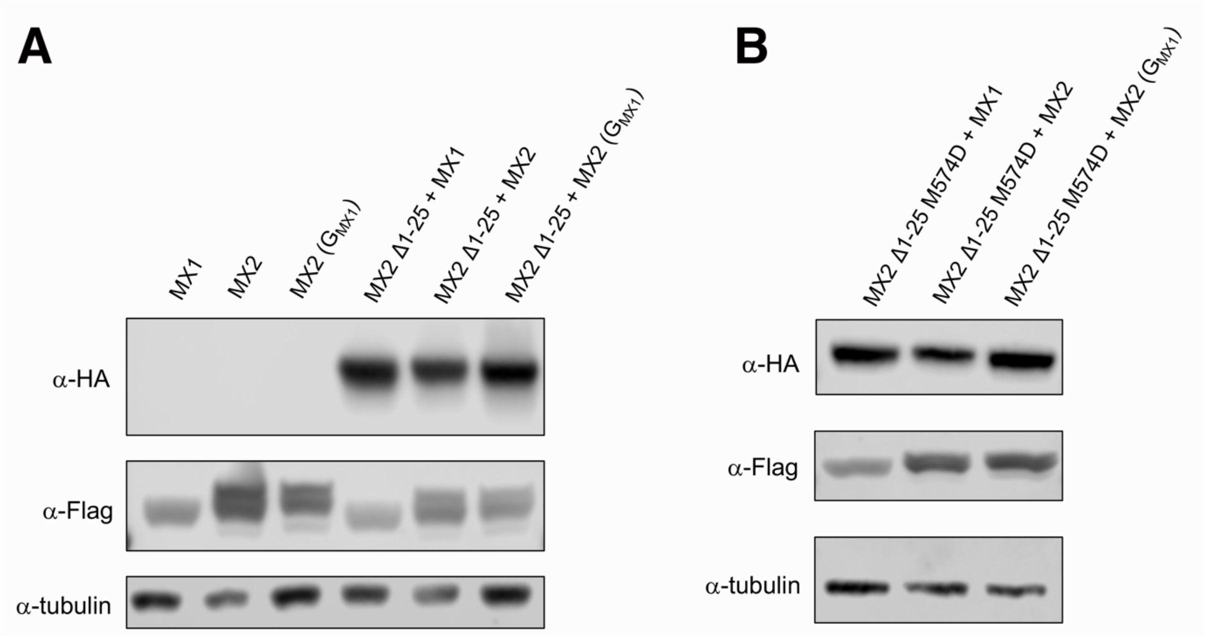

Figure S3. Protein levels of MX1, MX2, MX2 (G_{MX1}) and MX2 Δ1-25 in viral replication experiments (related to Figure 4B and C).

A Protein expression in cells transduced individually with Flag-tagged MX1, MX2 or MX2 (G_{MX1}), or together with HA-tagged MX2 Δ1-25 was analyzed by immunoblot, with α-tubulin included as the loading control.

B Protein expression in cells doubly transduced with Flag-tagged MX1, MX2 or MX2 (G_{MX1}), and HA-tagged MX2 Δ1-25 M574D was analyzed by immunoblot, with α-tubulin included as the loading control.

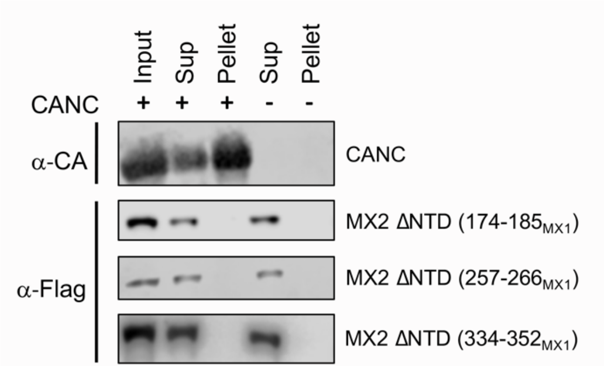

Figure S4. Identification of regions of the MX2 G domain important for CANC binding (related to Figure 5B).

MX2 Δ1-91 was mutated in positions 174-185, 257-266 or 334-352 by swapping MX2 residues for the corresponding residues of MX1. Lysates from expressing 293T cells were mixed with CANC assemblies, pelleted through a sucrose cushion, and Input, Sup and Pellet fractions were analyzed by immunoblot (n = at least 3). A representative immunoblot for CANC (α-CA) is shown.

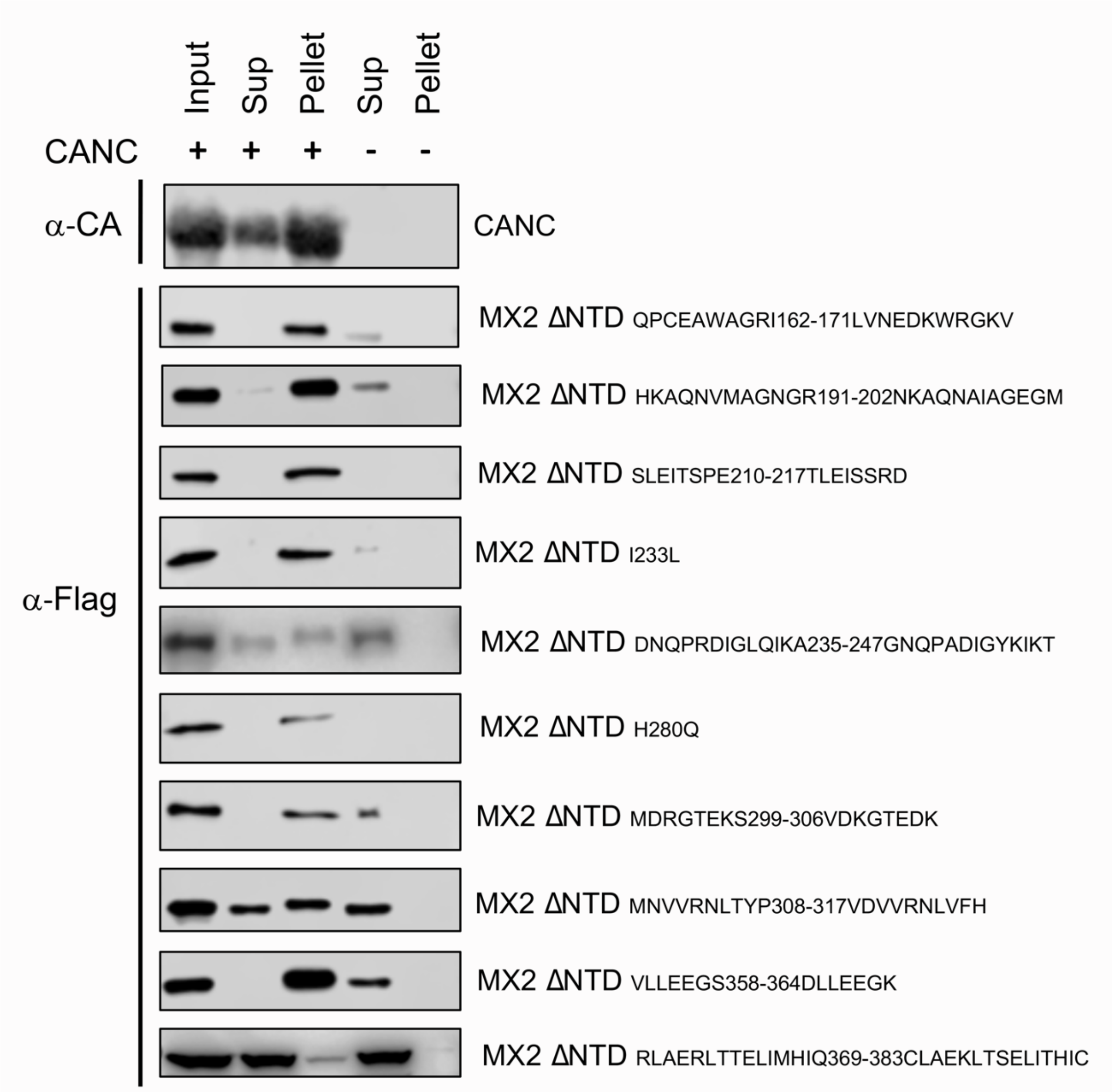

Figure S5. Characterization of regions in the MX2 G domain involved in the interaction with the viral capsid (related to Figure 5B).

Regions within the G domain of MX2 were swapped for the corresponding regions from MX1 in the MX2 ΔNTD background and tested for their ability to bind to assembled CANC complexes. Samples were pelleted through a sucrose cushion, and Input, Sup and Pellet fractions were analyzed by immunoblot (n = at least 3). A representative immunoblot for CANC (α-CA) is shown.

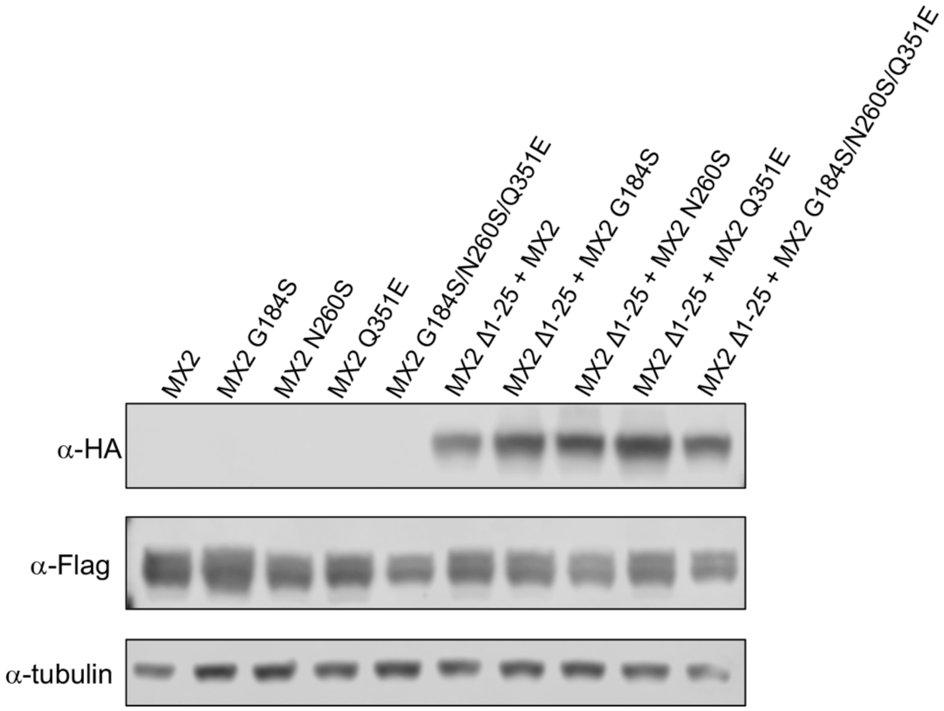

Figure S6. Protein levels of wild type MX2, mutants G184S, N260S, Q351E, G184S/N260S/Q351E and MX2 Δ1-25 in viral replication experiments (related to Figure 5D).

Protein expression in cells individually transduced with Flag-tagged MX2, MX2 G184S, MX2 N260S, MX2 Q351E or MX2 G184S/N260S/Q351E, or together with HA-tagged MX2 Δ1-25 was analyzed by immunoblot, with α-tubulin included as the loading control.

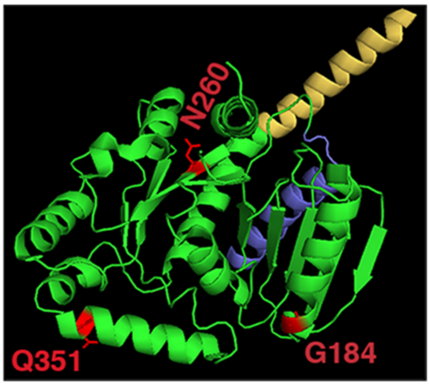

Figure S7. Structure of the G domain of MX2 (related to Figure 5C)

Crystal structure of the G domain of MX2 showing bundle signaling elements (BSE) 1 and 2, presented in ribbon diagrams. The G domain is shown in green, BSE 1 in blue and BSE 2 in red. The side chains of the three key residues identified as relevant for CANC binding are shown in red. The structure was drawn with the PyMOL molecular viewer (http://www.pymol.org). Atom coordinates were taken from PDB file 4WHJ (Fribough et al., 2014).
