# Supplementary material for: The GTPase Domain of MX2 Interacts with the HIV-1 Capsid, Enabling Its Short Isoform to Moderate Antiviral Restriction
Source: Cell Rep. 2019 Nov 12;29(7):1923–1933.e3. doi: 10.1016/j.celrep.2019.10.009 (PMC7391006; doi:10.1016/j.celrep.2019.10.009)
Supplement: Document S2. Article plus Supplemental Information [file mmc2.pdf]

Cell Reports

Article

# The GTPase Domain of MX2 Interacts with the HIV-1 Capsid, Enabling Its Short Isoform to Moderate Antiviral Restriction

## Graphical Abstract

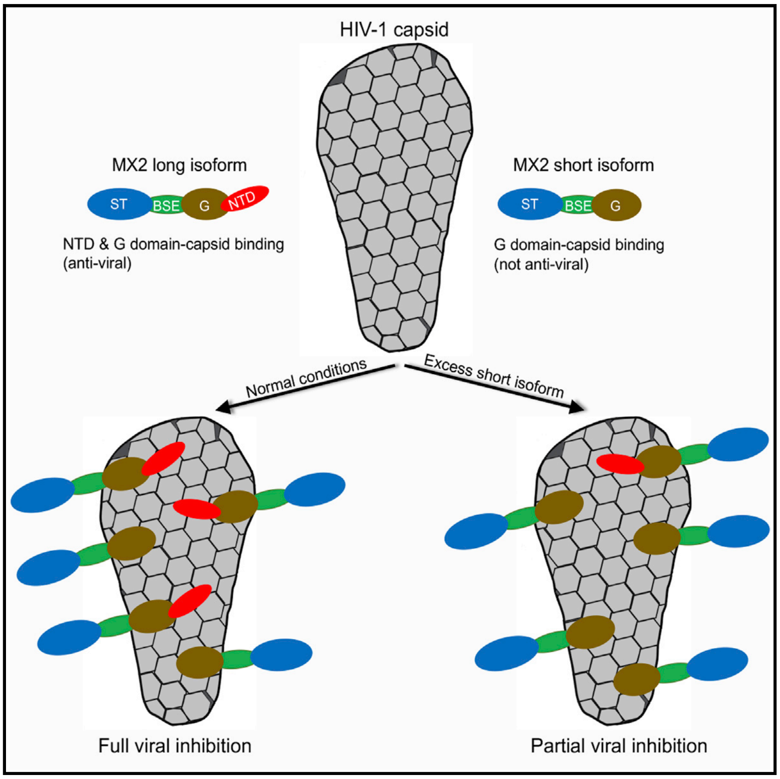


## Authors

Gilberto Betancor, Matthew D.J. Dicks, Jose M. Jimenez-Guardeño, Nabil H. Ali, Luis Apolonia, Michael H. Malim

## Correspondence

michael.malim@kcl.ac.uk

## In Brief

Myxovirus resistance 2 (MX2) inhibits HIV-1 infection by interacting with the viral capsid. Betancor et al. show that in addition to its N-terminal domain, MX2 binds to the capsid through its GTPase domain, therefore strengthening the interaction. Consequently, the non-antiviral short isoform competes with the long isoform for capsid binding and downmodulates viral inhibition.

## Highlights

- MX2 interacts with the HIV-1 capsid via N-terminal and GTPase (G) domains
- The G-domain interaction enhances MX2 binding to the viral capsid
- The MX2 short isoform is not antiviral and binds the capsid through its G domain
- The MX2 short isoform suppresses the antiviral activity of the long isoform

Betancor et al., 2019, Cell Reports *29*, 1923–1933
November 12, 2019 © 2019 The Author(s).
https://doi.org/10.1016/j.celrep.2019.10.009

CellPress

OPEN ACCESS
CellPress

# The GTPase Domain of MX2 Interacts with the HIV-1 Capsid, Enabling Its Short Isoform to Moderate Antiviral Restriction

Gilberto Betancor,[1] Matthew D.J. Dicks,[1,2] Jose M. Jimenez-Guardeño,[1,2] Nabil H. Ali,[1] Luis Apolonia,[1] and Michael H. Malim[1,3,*]
[1]Department of Infectious Diseases, School of Immunology & Microbial Sciences, King's College London, London SE1 9RT, UK
[2]These authors contributed equally
[3]Lead Contact
*Correspondence: michael.malim@kcl.ac.uk
https://doi.org/10.1016/j.celrep.2019.10.009

## SUMMARY

**Myxovirus resistance 2 (MX2/MXB) is an interferon (IFN)-induced HIV-1 restriction factor that inhibits viral nuclear DNA accumulation. The amino-terminal domain of MX2 binds the viral capsid and is essential for inhibition. Using *in vitro* assembled Capsid-Nucleocapsid (CANC) complexes as a surrogate for the HIV-1 capsid lattice, we reveal that the GTPase (G) domain of MX2 contains a second, independent capsid-binding site. The importance of this interaction was addressed in competition assays using the naturally occurring non-antiviral short isoform of MX2 that lacks the amino-terminal 25 amino acids. Specifically, these experiments show that the G domain enhances MX2 function, and the foreshortened isoform acts as a functional suppressor of the full-length protein in a G-domain-dependent manner. The interaction of MX2 with its HIV-1 capsid substrate is therefore multi-faceted: there are dual points of contact that, together with protein oligomerization, contribute to the complexity of MX2 regulation.**

## INTRODUCTION

Upon viral infection, activation of the innate immune response mobilizes an antiviral state. Central to this process is the production of type 1 interferons (IFNs); these cytokines induce the expression of a cascade of IFN-stimulated genes (ISGs), many of which have direct antiviral properties (Doyle et al., 2015; Bourke et al., 2018). As with many viruses, HIV type-1 (HIV-1) replication is suppressed by the IFN response (reviewed by Doyle et al., 2015). In recent years, human myxovirus resistance 2 (MX2; also called MXB) has been identified as a potent, IFN-induced inhibitor of HIV-1 infection (Goujon et al., 2013; Kane et al., 2013; Liu et al., 2013; Bulli et al., 2016; Dicks et al., 2018) and herpesvirus infection, including herpes simplex viruses 1 (HSV-1) and 2 (HSV-2) (Crameri et al., 2018; Schilling et al., 2018; Staeheli and Haller 2018). MX2 mediates a block to HIV-1 infection after reverse transcription (the synthesis of viral cDNA), but prior or together with the integration of viral DNA into host chromosomal DNA (Goujon et al., 2013; Kane et al., 2013; Liu et al., 2013; Matreyek et al., 2014).

Early studies showed that the HIV-1 Capsid (CA) protein is the virus-encoded determinant of MX2 sensitivity, since point mutations in CA can yield MX2-insensitive viruses (Goujon et al., 2013; Kane et al., 2013; Liu et al., 2013; Busnadiego et al., 2014; Matreyek et al., 2014; Bulli et al., 2016). Notably, such mutations may be located on different interfaces of the viral CA, as illustrated by the cyclophilin A (CYPA)-binding loop mutations P90A or G89V, and the hexamer trimeric-interface mutations G208R or T210K, thereby implying a complicated interplay between the CA and MX2. It has also been demonstrated that MX2 and the CA can interact *in vitro* using assemblies of the CA and CA-Nucleocapsid (CANC) as surrogates for the HIV-1 capsid lattice (Fribourgh et al., 2014; Fricke et al., 2014)

Because of the presence of an alternative start codon at position 26, human MX2 is expressed as two different isoforms of 78 and 76 kDa. Early work established that only the full-length form of MX2 is antiviral, while the short isoform has no observable anti-HIV-1 activity (Goujon et al., 2014; Matreyek et al., 2014), a feature shared in the inhibition of herpesviruses (Crameri et al., 2018; Schilling et al., 2018). Accordingly, it was proposed that only the long isoform can interact with CA (Fricke et al., 2014), with additional studies suggesting a dependence on the arginine residues at positions 11–13 within the amino-terminal domain (NTD) (Fricke et al., 2014; Schulte et al., 2015). Additionally, it has been shown that a chimeric dimer consisting of maltose-binding protein bearing the amino-terminal 35 residues of MX2 binds to CA assemblies, specifically at the CA tri-hexamer interface (Smaga et al., 2018). However, earlier work by Fribourgh et al. (2014) also showed that a truncated form of MX2 lacking the amino-terminal 84 residues may still interact with CA assemblies, albeit with lower affinity. Another unresolved aspect of MX2's mechanism of action concerns the extent of oligomerization needed for viral inhibition, since some studies have found that MX2 dimerization is sufficient (Buffone et al., 2015; Dicks et al., 2015), whereas another analysis proposed that higher-order oligomers might be important (Alvarez et al., 2017).

Human MX2 belongs to the dynamin-like GTPase family, which also includes the related protein MX1 (also called MXA). MX1 has been acknowledged as an antiviral ISG for many years (reviewed by Verhelst et al., 2013) and can act on a broad


This is an open access article under the CC BY license (http://creativecommons.org/licenses/by/4.0/).

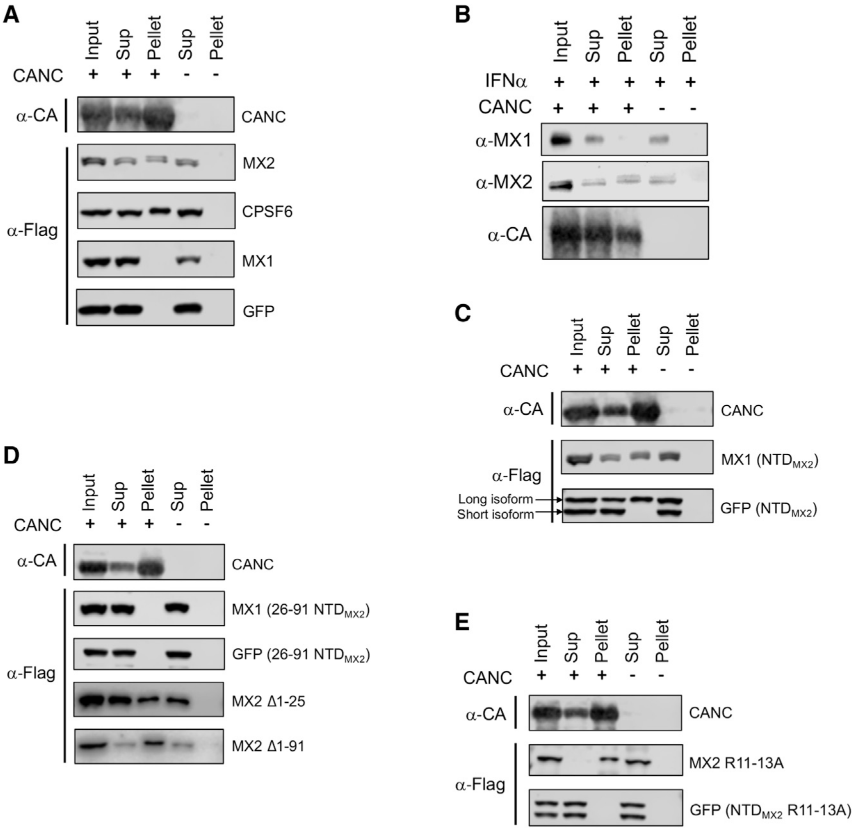


**Figure 1. Binding of MX2 and Chimeric Proteins to HIV-1 CANC Nanotubes**

(A) 293T cells were transfected with vectors expressing human MX2, human MX1, GFP, or human CPSF6. Cell lysates were then incubated in the presence or absence of *in vitro* assembled CANC complexes and subjected to centrifugation through a sucrose cushion. Resulting supernatant (Sup) and pellet fractions, together with the input sample, were analyzed by immunoblot (n ≥ 20). A representative immunoblot for CANC (α-CA) is shown.

(B) CANC complexes were incubated with lysates from U87-MG CD4/CXCR4 cells treated with 500 U/ml of IFNα and pelleted through a sucrose cushion. Input, Sup, and pellet fractions were analyzed by immunoblot (n = 4).

(C) Chimeric MX1 or GFP proteins containing the N-terminal domain of MX2 were expressed in 293T cells. Cell lysates were incubated with CANC complexes, pelleted through a sucrose cushion, and input, Sup, and pellet fractions were analyzed by immunoblot. A representative immunoblot for CANC (α-CA) is shown (n = 5).

(D) Deletions of residues 1–25 were introduced into MX2, MX1 (NTD$_{MX2}$), or GFP (NTD$_{MX2}$), as well as deletion of residues 1–91 from MX2. 293T cell lysates expressing these proteins were mixed with CANC assemblies, pelleted through a sucrose cushion, and input, Sup, and pellet fractions were analyzed by immunoblot (n ≥ 4). A representative immunoblot for CANC (α-CA) is shown.

(E) The triple-arginine mutation R11-13A was introduced into MX2 and GFP (NTD$_{MX2}$). Lysates from transfected 293T cells expressing these proteins were mixed with CANC assemblies, pelleted through a sucrose cushion, and input, Sup, and pellet fractions were analyzed by immunoblot (n = 5). A representative immunoblot for CANC (α-CA) is shown.

spectrum of viruses, including influenza A virus (IAV), hepatitis B virus (HBV), Thogoto virus (THOV), or measles virus (Pavlovic et al., 1990; Frese et al., 1996; Kochs and Haller, 1999; Gordien et al., 2001), but not HIV-1 (Goujon et al., 2013; Kane et al., 2013; Liu et al., 2013). Like MX1, MX2 is composed of a disordered NTD, followed by a GTPase (G) domain and a stalk (ST) domain, connected by three hinge-like bundle signaling elements (BSEs). While the G and ST domains of MX1 and MX2 are notably similar in sequence and overall structure (Gao et al., 2010, 2011; Fribourgh et al., 2014), the NTD of MX2 is notably longer than that of MX1 (91 versus 43 amino acids, respectively). There are clear differences in the determinants of viral inhibition between MX1 and MX2: (1) While MX2 prevents the nuclear accumulation of HIV-1 cDNAs, human MX1 is thought to act at a post-transcriptional level by inhibiting the nuclear export of HBV RNAs (Gordien et al., 2001) or prior to transcription by trapping THOV nucleocapsids in the cytoplasm (Kochs and Haller, 1999). (2) A disordered loop in the ST domain (called L4) of MX1 is an important determinant of virus inhibition, at least for IAV and THOV, where an interaction with the viral nucleoprotein (NP) has been shown (Mitchell et al., 2012), but this element is dispensable for MX2-mediated HIV-1 suppression (Goujon et al., 2014; Verhelst et al., 2015). (3) For MX2, a key region necessary for the inhibition of HIV-1 has been shown to be the NTD; for example, a chimeric MX1 protein bearing the NTD of MX2 displays equivalent antiviral activity to wild-type MX2 (Goujon et al., 2014, 2015). (4) While GTPase activity is required for MX1-mediated virus suppression, this enzymatic capability is dispensable for HIV-1 inhibition by MX2 (Mitchell et al., 2012; Goujon et al., 2013; Kane et al., 2013; Matreyek et al., 2014). However, GTP hydrolysis may be involved in the organization of MX2 oligomerization (Alvarez et al., 2017), and GTPase activity is required for the inhibition of herpesvirus infections (Crameri et al., 2018; Schilling et al., 2018).

Here, we investigate in depth the requirements for the interaction between MX2 and the HIV-1 capsid. We show that in addition to the previously described NTD binding site, the G domain of MX2 encodes a second, independent capsid-binding site. Importantly, this site not only contributes to the antiviral activity of full-length MX2, but it also enables the short MX2 isoform to act as a negative regulator of the full-length protein.

## RESULTS

### MX2 Binds Assembled HIV-1 CANC Complexes

The HIV-1 capsid lattice that forms the distinctive conical core of viral particles is composed of hexameric and pentameric assemblies of the viral CA protein. One well-established experimental model for studying the capsid lattice entails the use of CANC nanotubes that are assembled *in vitro* using

**Table 1. Summary of MX1/MX2 Chimeric Protein Binding to CANC Complexes**

| MX1/MX2 Chimera | CANC binding? |
|---|---|
| MX1 ($L4_{MX2}$) | No |
| MX1 ($G_{MX2}$) | No |
| MX1 ($ST_{MX2}$) | No |
| MX1 (G, $ST_{MX2}$) | No |
| MX1 (BSE1-$3_{MX2}$) | No |
| MX1 ΔNTD | No |
| MX2 ($NTD_{MX1}$) | Low |
| MX2 ΔNTD (G, $ST_{MX1}$) | No |
| MX2 ΔNTD ($G_{MX1}$) | No |
| MX2 ΔNTD ($ST_{MX1}$) | Yes |
| MX2 ΔNTD ($L4_{MX1}$) | Yes |
| MX2 ΔNTD | Yes |
| MX1 ΔNTD ($G_{MX2}$) | Yes |

purified recombinant protein (Campbell and Vogt, 1995; Gross et al., 1997). These have been employed extensively for high-resolution structural studies (Bharat et al., 2012; Dick et al., 2018) and for analyzing interactions between the capsid lattice and cellular proteins (Valle-Casuso et al., 2012; Li and Sodroski, 2008; Black and Aiken, 2010; Dharan et al., 2017; Schaller et al., 2017).

It has previously been reported that human MX2 interacts with HIV-1 CANC complexes (Fribourgh et al., 2014; Fricke et al., 2014). We first confirmed this observation using extracts of transfected 293T cells expressing MX1 or MX2, as well as a known CA-binding protein (cleavage and polyadenylation specificity factor subunit 6 [CPSF6]; Price et al., 2012) and an unrelated negative control protein (green fluorescent protein, GFP), all bearing a FLAG-tag. Cell lysates were incubated with assembled CANC complexes, subjected to centrifugation through a sucrose cushion such that only very-high-molecular-weight CANC complexes together with interacting factors were pelleted, and then analyzed by immunoblotting.

As expected, CPSF6 was readily detected in the pellet fraction when CANC complexes were present, but not when they were absent, and GFP failed to interact with these complexes (Figure 1A). As previously shown (Fricke et al., 2014), MX2 was also able to bind to CANC complexes, while the related MX1 protein was not, again illustrating the specificity of this interaction (Figure 1A). Further confirmation of this finding was obtained using lysates from IFNα-treated U87-MG CD4/CXCR4 cells, where both the long and short isoforms of endogenous IFNα-induced MX2 were shown to interact with CANC complexes, but endogenous MX1 did not (Figure 1B) (Fricke et al., 2014).

## The NTD of MX2 Is Not Solely Responsible for the Interaction with the HIV-1 Capsid Lattice

Previous studies have proposed that the NTD of MX2 is entirely responsible for capsid binding (Fricke et al., 2014; Schulte et al., 2015), though other analyses have indicated that this region may not be absolutely required (Fribourgh et al., 2014). To explore the capsid-binding determinants of MX2 in further detail, we determined the CANC binding phenotypes for a series of GFP/MX2 and MX1/MX2 chimeric proteins, as well as MX2 deletion and point mutants. We first transferred the NTD of MX2 to MX1 and GFP, with the resulting chimeric proteins (MX1 [$NTD_{MX2}$] and GFP [$NTD_{MX2}$]) naturally expressing corresponding long and short isoforms (Figure 1C) due to the alternative start codon at position 26. As noted before, MX1 carrying the MX2 NTD acquired the ability to bind to CANCs (Fricke et al., 2014), an observation recapitulated with the chimeric GFP ($NTD_{MX2}$) protein (Figure 1C); importantly, only the chimera corresponding to the long isoform of MX2 bound to CANC assemblies, with the chimera

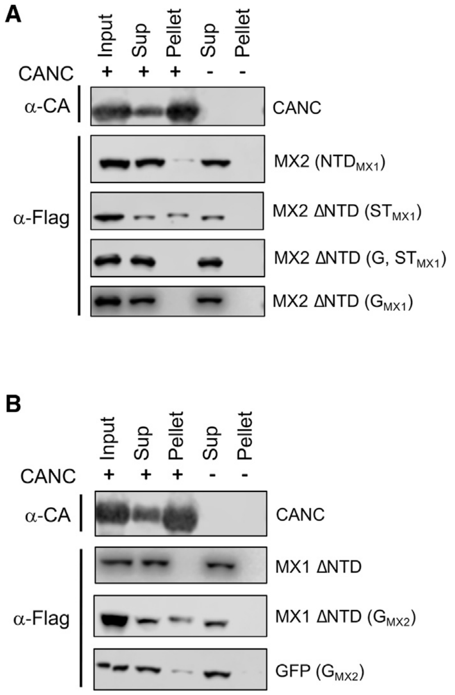


**Figure 2. MX2 Is Able to Bind to CANC Complexes through Its G Domain**

(A) NTD, ST, and/or G domains of MX1 were introduced into the truncated form of MX2 lacking the NTD (MX2 ΔNTD). 293T cell lysates expressing these proteins were mixed with CANC assemblies, pelleted through a sucrose cushion, and input, Sup, and pellet fractions were analyzed by immunoblot (n = 5). A representative immunoblot for CANC (α-CA) is shown.

(B) NTD truncated MX1 (MX1 ΔNTD) with or without the G domain of MX2, or a chimeric protein comprising GFP and the G domain of MX2 were expressed in 293T cells. Cell lysates were mixed with CANC assemblies, pelleted through a sucrose cushion, and input, Sup, and pellet fractions were analyzed by immunoblot. A representative immunoblot for CANC (α-CA) is shown (n ≥ 3).

corresponding to the short isoform that lacks the amino-terminal 25 residues (GFP [$NTD_{MX2}$ Δ1-25]) failing to interact (Figure 1C).

Next, focusing on the NTD, we found that the deletion of the amino-terminal 25 amino acids from the GFP ($NTD_{MX2}$) and MX1 ($NTD_{MX2}$) chimeric proteins (i.e., proteins that correspond to short isoforms expressed from the downstream codon) abolished the ability of the NTD to interact with CANC complexes (Figure 1D). Critically, however, we found that the short isoform of MX2 (MX2 Δ1-25) was still able to bind to CANC complexes (Figure 1D), consistent with previous results using recombinant MX2 that lacked the first 84 residues (Fribourgh et al., 2014). This suggested that MX2 contains capsid-binding determinants that reside beyond the amino-terminal 84 amino acids; this was reinforced by the finding that a truncated form of MX2 lacking the entire NTD (MX2 Δ1-91; hereafter called MX2 ΔNTD) also interacted with CANC complexes (Figure 1D). Residues 11–13 of the NTD of MX2 have been shown to be important for both viral inhibition (Goujon et al., 2015) and CANC complex binding (Schulte et al., 2015; Smaga et al., 2018). While substitution of these three arginines for alanine abrogated the interaction between GFP ($NTD_{MX2}$) and CANC complexes, the effect on the binding of full-length MX2 was modest (Figure 1E). Taken together, these data confirm the important role played by the triple-arginine motif of the NTD in CA binding and highlight the importance of the additional, independent region(s) of MX2 for interacting with the HIV-1 capsid.

## The G Domain of MX2 Interacts with the HIV-1 Capsid

To map the additional region(s) of MX2 that can bind the capsid lattice, we created a library of MX1/MX2 chimeras where different protein domains were swapped and binding to CANC complexes was assessed (Table 1). One unexpected complication in these experiments was that the NTD of MX1 appeared to have an inhibitory role on the ability of MX2 to bind to CANCs (Table 1; Figure 2A). Accordingly, all chimeric proteins where MX1 domains were introduced into MX2 were configured not to contain an NTD. We found that exchanging the MX2 G domain either alone or with the ST domain for corresponding regions of MX1 abrogated CANC complex binding (Figure 2A). Importantly, a reverse chimera comprising MX1 ΔNTD bearing the G domain of MX2 clearly interacted with CANC assemblies, whereas MX1 ΔNTD did not (Figure 2B). Finally, by showing that a fusion of the MX2 G domain to GFP, GFP ($G_{MX2}$), also bound to CANCs (albeit relatively modestly), we confirmed that the G domain of MX2 contains a second capsid-binding site (Figure 2B).

## The Short Isoform of MX2 (MX2 Δ1-25) Negatively Regulates Wild-Type MX2 Function

Having demonstrated that the short isoform of MX2, which lacks antiviral activity by itself (Goujon et al., 2014; Matreyek et al., 2014), is able to bind to the HIV-1 capsid lattice (Figure 1D), we next asked whether it might play a role in modulating the antiviral function of full-length MX2. Since both isoforms of MX2 are IFN inducible, we tested the ability of the short isoform to inhibit HIV-1 infection when ectopically expressed in the absence of IFNα or in its presence (i.e., when both endogenous isoforms are expressed). As controls, we also ectopically expressed wild-type MX2 (i.e., both isoforms) or an irrelevant protein (luciferase; negative control).

In luciferase-expressing cells, the addition of IFNα yielded the expected drop (~16-fold) in viral infection (Figure 3A). As a positive control, overexpression of MX2 severely reduced viral infectivity in both the presence and the absence of IFNα compared to the luciferase-expressing cells. As previously established, expression of the short isoform of MX2 alone had no effect on the efficiency of infection. In contrast, however, this protein significantly diminished the inhibitory effect of IFNα: ~6-fold inhibition, compared to ~16-fold for the luciferase control (Figure 3A). Critically, this result suggests that the short isoform of MX2 can counteract the IFN-induced suppression of HIV-1, with one natural possibility being that it interferes with the activity of the long MX2 isoform. Such interference could be due to two main effects: (1) the short isoform competes with the long isoform for CA binding, or (2) overexpression of the short isoform alters the oligomerization equilibrium between the two MX2 isoforms, thereby perturbing antiviral activity. To distinguish between these scenarios, we overexpressed monomeric MX2 Δ1-25 bearing the M574D mutation (Buffone

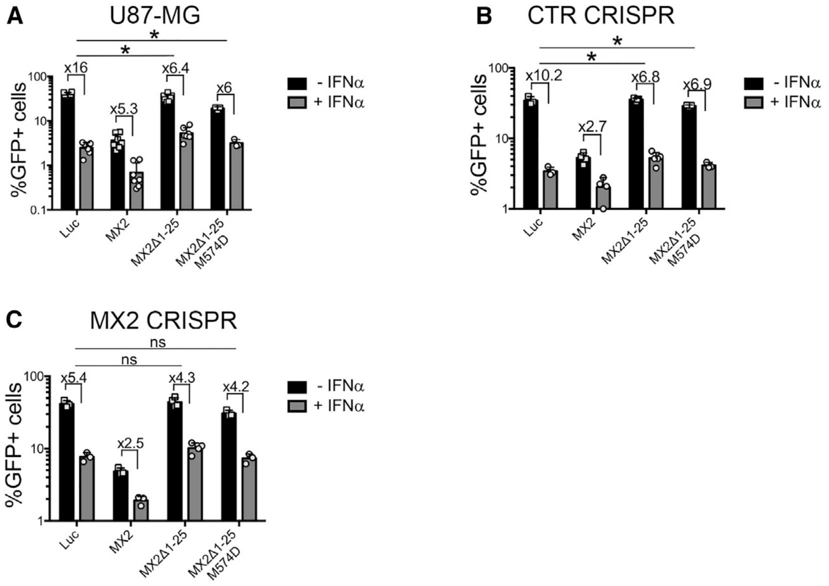


**Figure 3. The Short Isoform of MX2 Is a Dominant Negative Regulator of the Long Isoform in HIV-1-Infected Cells**

(A) U87-MG CD4/CXCR4 cells were transduced with EasiLV-expressing luciferase (Luc), MX2, MX2 Δ1-25, or MX2 Δ1-25 M574D and treated with doxycycline for 24 h. At that time, half of the cells were treated with 500 U/ml IFNα for a further 24 h while the other half was left untreated. Cells were then challenged with an HIV-1-based lentiviral vector expressing GFP. After 2 days, the percentage of GFP-expressing cells was evaluated by flow cytometry (n ≥ 3, mean ± SD). The fold-difference in infection in the presence of IFNα is indicated (*p < 0.05, unpaired t test). See also Figure S1.

(B and C) As in (A), but using CTR CRISPR (B) or MX2 CRISPR (C) U87-MG CD4/CXCR4 cells (n ≥ 3, mean ± SD). The fold-difference in infection in the presence of IFNα is indicated (*p < 0.05; ns, non-significant; unpaired t test).

See also Figure S1.

et al., 2015; Dicks et al., 2015), excluding any possibility of interaction between both protein isoforms. Critically, the IFN-imposed block was reduced to a similar extent as seen with MX2 Δ1-25, indicating that competing for CA binding rather than altered oligomerization underlies the interference with MX2 activity.

To further characterize the inhibition of the IFN-imposed block induced by the short isoform of MX2, we used an engineered U87-MG CD4/CXCR4-derived cell line where the MX2 alleles had been inactivated using CRISPR-Cas9 technology (Dicks et al., 2018), as well as a control (CTR) CRISPR cell line. Importantly, the reductions in IFN-induced virus suppression seen when MX2 Δ1-25 or MX2 Δ1-25 M574D are expressed were only observed in the CTR CRISPR cells and not in MX2 CRISPR cells, demonstrating that the endogenous MX2 protein is the specific molecular target of this effect (Figures 3B and 3C; Figure S1 shows the corresponding immunoblot analyses of protein expression level).

## The G-domain-Capsid Interaction Enhances the Antiviral Activity of MX2

In seeking to substantiate our model for MX2 isoforms competing for capsid binding, we first returned to the CANC interaction assay. More specifically, we used a competition assay to focus on whether the G domain of MX2 affects binding to CANC complexes in the context of mixed reactions where full-length wild-type MX2 was also present. We therefore incubated CANCs with extracts containing full-length MX2 bearing an HA (hemagglutinin)-tag, plus either wild-type MX2, MX2 R11-13A, MX2 ($G_{MX1}$), or MX1 ($NTD_{MX2}$), each carrying a FLAG-tag. Levels of pelleted MX2-HA and FLAG-tagged proteins were compared to measure how well each MX variant competes against wild-type MX2 for CANC binding; MX2-FLAG served as the control reaction, and all values for relative binding were compared to this sample. As shown in Figure 4A, MX proteins that interact with capsid via a single region—either the G domain (MX2 R11-13A) or the NTD (MX2 ($G_{MX1}$) and MX1 ($NTD_{MX2}$)—all competed for CANC binding less effectively than wild-type MX2, with relative binding only reaching ~50%–60% of that of the wild-type protein. Importantly, these reductions in binding capacity were similar in degree irrespective of whether the NTD or the G domain was missing, highlighting the significant role played by both domains in maximizing efficient capsid binding.

Though these results demonstrate the increased capsid association of wild-type MX2 compared to MX2 ($G_{MX1}$), we have previously reported that this chimeric protein is as antiviral as wild-type MX2 in single-cycle infection experiments (Goujon et al., 2014). To gain further insight into this issue, we performed spreading HIV-1 infection experiments in the presence of MX2, MX2 ($G_{MX1}$), or MX1 (as negative control) alone or together with the short isoform of MX2. When alone, MX2 and MX2 ($G_{MX1}$) sustained the same viral replication kinetics, at least during the first 3 days of infection, followed by a slightly lower viral production for MX2 (Figure 4B, left panel), consistent with previous results (Goujon et al., 2014). Importantly, when co-expressed with MX2 (Δ1-25), MX2 displayed stronger antiviral function than MX2 ($G_{MX1}$) (Figure 4B, right panel), thus confirming the impaired antiviral capacity of MX2 containing the G domain of MX1. In an independent validation, a similar phenotype was also observed in single-cycle experiments where cells were challenged with an HIV-1-based lentiviral vector (Figure S2A). Corresponding immunoblot analyses of protein expression levels are shown in Figure S3A.

We and others have demonstrated the importance of MX2 oligomerization for antiviral activity (Buffone et al., 2015; Dicks et al., 2015; Alvarez et al., 2017). To rule out a role for oligomerization in these results, we performed the same competition experiments described in Figure 4B in the presence of the monomeric short isoform MX2 Δ1-25 M574D. Results obtained show how under these conditions, where no interaction between the short isoform and wild-type/chimeric MX2 is possible, wild-type MX2 again restricted HIV-1 more effectively than MX2

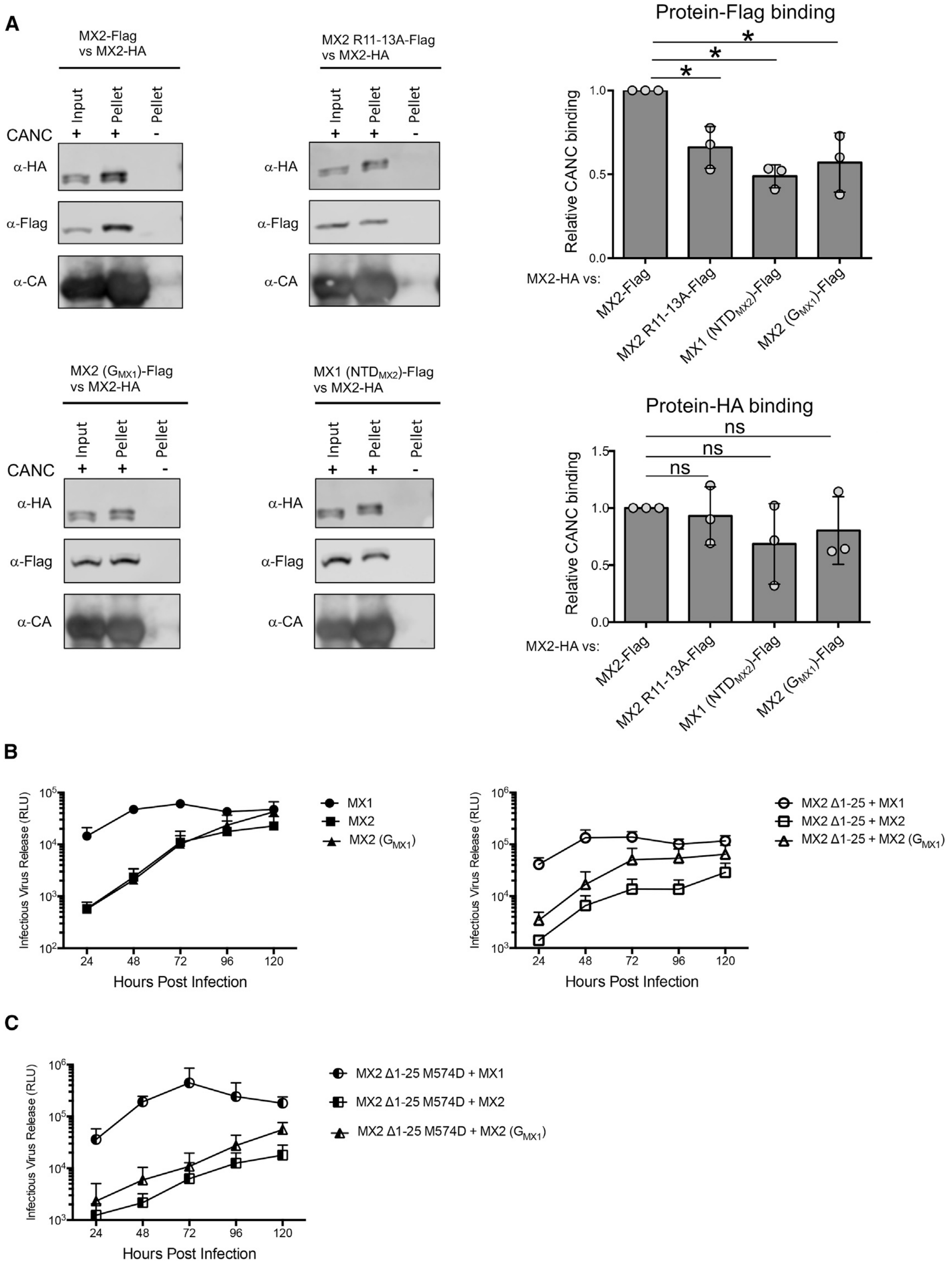


**Figure 4. The G Domain of MX2 Enhances the Interaction with CANC Complexes and HIV-1 Inhibition**

(A) Cell lysates from 293T cells expressing HA-tagged wild-type MX2 were mixed with cell lysates expressing FLAG-tagged wild-type MX2, MX2 R11-13A, MX2 ($G_{MX1}$), or MX1 ($NTD_{MX2}$). These were added to CANC assemblies, pelleted through a sucrose cushion, and the HA- and FLAG-tagged proteins present in input, Sup, and pellet fractions were quantified by immunoblot and corrected for the amount of protein present in the input. The level of bound protein in each competition experiment was normalized to the value obtained in the MX2-HA versus MX2-FLAG sample (n = 3, mean ± SD; *$p < 0.05$; ns, non-significant; unpaired t test).

*(legend continued on next page)*

($G_{MX1}$) (Figure 4C; Figure S3B shows the corresponding immunoblot analyses). A similar phenotype was also obtained in single-cycle experiments using an HIV-1 lentiviral vector (Figure S2B).

### The Interaction between the G Domain of MX2 and the Capsid Is Complex

In order to map the region(s) of the G domain important for the interaction with capsid, we created three chimeric MX2 ΔNTD proteins where G-domain residues 92–234, 235–322, and 323–387 (i.e., spanning the whole G domain) were swapped for their complementary regions from MX1 (MX2 ΔNTD [$92\text{-}234_{MX1}$], MX2 ΔNTD [$235\text{-}322_{MX1}$], and MX2 ΔNTD [$323\text{-}387_{MX1}$], respectively). None of these chimeric proteins were able to bind to CANC assemblies (Figure 5A), suggesting the involvement of all three regions in the interaction.

Alignment of the G-domain sequences of MX1 and MX2 shows 13 regions where the amino acid sequences differ (Figure 5B, underlined residues). We therefore introduced each region individually into MX2 ΔNTD and addressed their CANC-binding ability. Interestingly, we found that three discrete regions are necessary for CANC binding (residues 174–185, 257–266, and 334–352; highlighted in gray in Figures 5B and S4), while all other mutant proteins maintained their ability to bind (Figure S5). Of note, each of these three regions is located within one of the extended regions analyzed in Figure 5A.

We then individually mutated all residues within these three regions and found that amino acids G184, N260, and Q351 are necessary for the G domain's interaction with the capsid (Figure 5C; indicated in bold within each highlighted region in Figure 5B). Finally, we introduced the G184S, N260S, and Q351E mutations individually or in combination into full-length MX2 and performed competition experiments against MX2 Δ1-25 during spreading HIV-1 replication. Cells expressing wild-type MX2 sustained lower levels of viral replication than seen with any of the mutants, particularly when compared to the triple mutant G184S/N260S/Q351E (Figure 5D; Figure S6 shows the corresponding immunoblot analyses). In the absence of MX2 Δ1-25, none of these mutants supported higher viral replication when compared to wild-type MX2 (data not shown), as expected from prior results obtained under the same conditions with MX2 ($G_{MX1}$) (Figure S2A). These results demonstrate that multiple residues within MX2's G domain contribute to capsid binding and confirm their importance for full antivirus function.

### Oligomerization Increases the Avidity of the MX2-Capsid Interaction

We and others have shown how MX2 dimerization, but not higher-order oligomerization, is required for viral inhibition (Buffone et al., 2015; Dicks et al., 2015), indicating that the monomeric protein lacks antiviral activity. Therefore, we introduced the monomer-only mutations M574D and M527D into MX2 and MX1 ($NTD_{MX2}$), respectively, and examined their ability to bind to assembled CANC complexes (Figure 6A). In agreement with previous work, dimerization or higher-order oligomerization was not required for CANC interaction (Buffone et al., 2015), although the levels of protein recovered in the pellet fraction were visibly reduced. To quantify the contribution of MX2 oligomerization to capsid binding, we then performed *in vitro* competition experiments. We incubated CANC assemblies with FLAG-tagged MX2 M574D together with HA-tagged wild-type MX1, MX2, or MX2 M574D, and the levels of pelleted proteins were quantified. Inspection of the data shows that the binding of MX2 M574D-FLAG to CANCs was most efficient in the absence of competition (i.e., in the presence of MX1-HA). Importantly, the binding of MX2 M574D-FLAG was reduced more effectively in competition with wild-type MX2-HA, compared to competition against MX2 M574D-HA (Figure 6B; compare left and middle panels). This result demonstrates the importance of protein oligomerization for increasing the avidity of MX2-capsid binding.

## DISCUSSION

The G domain of MX1 plays an important role in the inhibition of different viruses, such as IAV and THOV (Pitossi et al., 1993; Ponten et al., 1997). Similarly, it has recently been described that the GTPase activity of MX2 is necessary for the inhibition of a number of herpesviruses (Crameri et al., 2018; Schilling et al., 2018). However, the role of the G domain of MX2 in the inhibition of HIV-1 has been less clear, particularly as mutant proteins deficient for GTPase enzymatic activity maintain antiviral activity (Goujon et al., 2013; Kane et al., 2013; Matreyek et al., 2014).

In this work, we revisit the importance of the MX2 G domain for the control of HIV-1 infection. We show how, in addition to the MX2-capsid interaction that is driven by the NTD, MX2 also interacts with the capsid through its G domain. CANC-interaction experiments using chimeric proteins demonstrate that transfer of the G domain of MX2 to MX1 ΔNTD or GFP is sufficient to confer CANC binding (Figure 2B), thus defining a second point of interaction in addition to that mediated by the MX2 NTD (Figure 1C). *In vitro* competition experiments further support these findings and demonstrate that the G domain enhances the interaction between MX2 and the HIV-1 capsid lattice, a point that is illustrated by wild-type MX2 outcompeting MX2 ($G_{MX1}$) for binding to HIV-1 CANC complexes (Figure 4A). While the contributions of G domains to MX-mediated viral suppression are well recognized (Pitossi et al., 1993; Gao et al., 2010; Crameri et al., 2018;

(B) U87-MG CD4/CXCR4 cells were transduced with a lentiviral vector system conferring puromycinR and expressing FLAG-tagged MX1, MX2, or MX2 ($G_{MX1}$) alone or together with the EasiLV-expressing HA-tagged MX2 Δ1-25. Two days later, transduced cells were selected with 1 μg/ml puromycin for 48 h and MX2 Δ1-25 induced with 0.5 μg/ml doxycycline. Cells were then infected with HIV-$1_{NL4\text{-}3}$ (185 ng $p24^{Gag}$), and culture supernatants were harvested and stored every 24 h. Infectivity of each collected sample was determined using HeLa-TZMbl indicator cells and measuring chemiluminescent β-galactosidase activity at 48 h (n = 4, mean ± SD). See also Figures S2A and S3.

(C) As in (B), but expressing HA-tagged monomeric mutant MX2 Δ1-25 M574D together with MX1, MX2, or MX2 ($G_{MX1}$), instead of MX2 Δ1-25 (n = 4, mean ± SD). See also Figures S2B and S3.

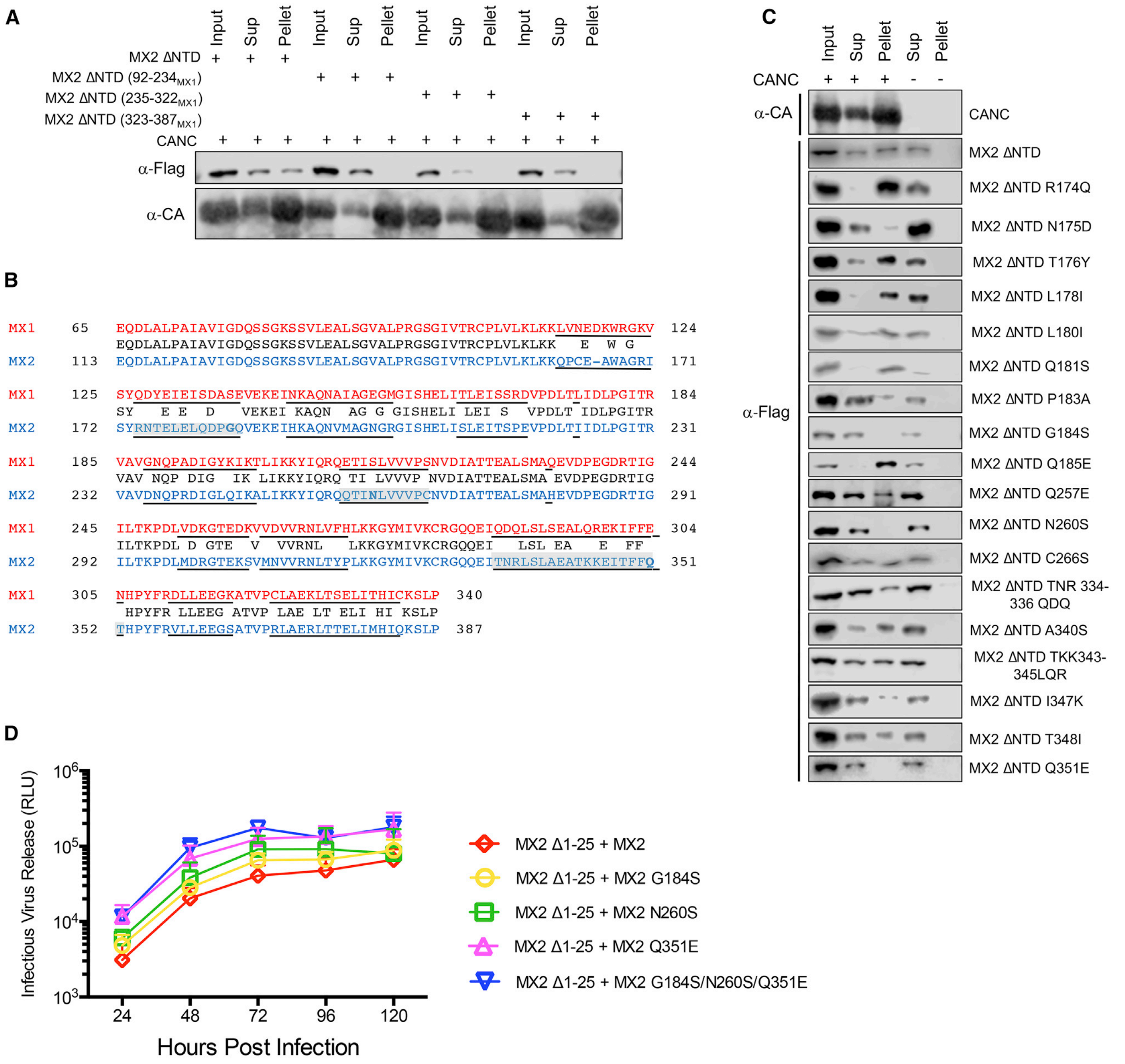

**Figure 5. Identification of Single Residues Important for the Capsid-G-Domain Interaction and the Antiviral Function of MX2**

(A) Chimeric proteins MX2 ΔNTD (92-234$_{MX1}$), MX2 ΔNTD (235-322$_{MX1}$), and MX2 ΔNTD (323-387$_{MX1}$) were expressed in 293T cells and lysates mixed with CANC assemblies, pelleted through a sucrose cushion. input, Sup, and pellet fractions were analyzed by immunoblot (n = 4).

(B) Sequence alignment of the G-domain regions of human MX1 (red) and MX2 (blue) showing the consensus sequence between them (black). Underlined are the 13 regions of sequence difference, with the three regions found to be important for CANC binding highlighted and the three key residues shown in bold. See also Figure S4.

(C) Residues found on the three different regions of the MX2 G domain important for CANC binding were individually mutated to their MX1 counterparts in a MX2 ΔNTD background and the proteins expressed in 293T cells. Lysates were mixed with CANC assemblies, pelleted through a sucrose cushion, and input, Sup, and pellet fractions were analyzed by immunoblot (n ≥ 3). A representative immunoblot for CANC (α-CA) is shown. See also Figure S7.

(D) U87-MG CD4/CXCR4 cells doubly transduced with puromycinR lentiviral vectors expressing MX2, MX2 G184S, MX2 N260S, or MX2 Q351E together with EasiLV-expressing MX2 Δ1-25 were selected with 1 μg/ml puromycin and treated with 0.5 μg/ml doxycycline to induce MX2 Δ1-25. Cells were infected with HIV-1$_{NL4-3}$ (350 ng p24$^{Gag}$), and culture supernatants were harvested every 24 h. Levels of infectivity for each sample were determined using HeLa-TZMbl indicator cells (n = 4, mean ± SD).

See also Figure S6.

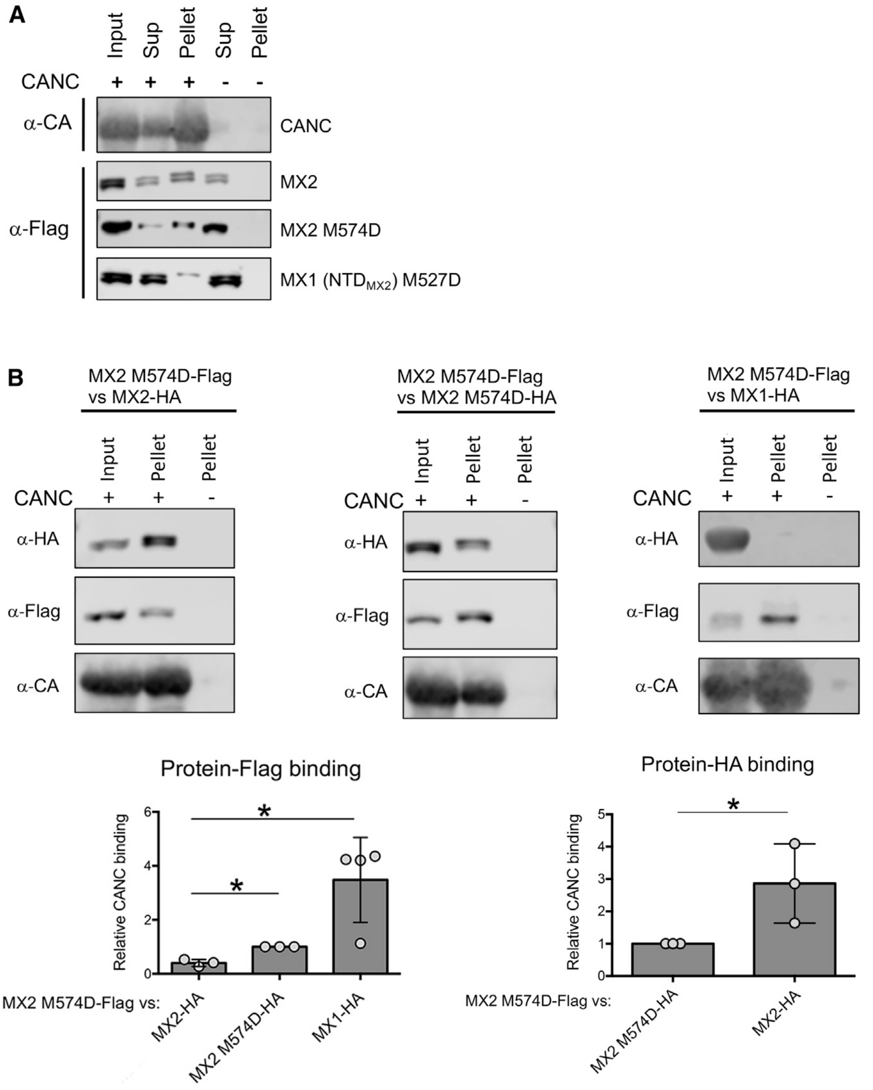


**Figure 6. Oligomerization of MX2 Increases the Avidity for Capsid**

(A) 293T cells were transfected with vectors expressing wild-type MX2 or monomeric mutants MX2 M574D and MX1 ($NTD_{MX2}$) M527D. Lysates were mixed with CANC assemblies, pelleted through a sucrose cushion, and input, Sup, and pellet fractions were analyzed by immunoblot ($n \geq 3$). A representative immunoblot for CANC (α-CA) is shown.

(B) Cell lysate from 293T cells expressing FLAG-tagged MX2 M574D was mixed with lysates expressing HA-tagged wild-type MX1, MX2, or MX2 M574D. Mixtures were added to CANC assemblies, pelleted through a sucrose cushion, and the HA- and FLAG-tagged proteins present in the input and pellet fractions quantified by immunoblot. The ability of each protein to bind to CANC was calculated as the ratio between the band intensities measured in pellet and input fractions. Finally, their relative CANC binding activity was determined by comparing the values with those obtained for MX2 M574D-FLAG versus MX2 M574D-HA (n = 3, mean ± SD; $^*p < 0.05$; unpaired t test).

Schilling et al., 2018), we now present evidence for a G domain also acting through direct binding to a viral substrate, rather than by catalyzing GTP hydrolysis.

Working from this, we have shown that the short isoform of MX2 (MX2 Δ1-25), by virtue of its intact G domain, can therefore interact with the capsid. Though it lacks antiviral function (Goujon et al., 2013; Kane et al., 2013; Fricke et al., 2014; Matreyek et al., 2014), this result prompted us to revisit the functionality of MX2 Δ1-25 during the IFN-induced inhibition of HIV-1 infection (when this form is naturally expressed; Goujon et al., 2013; Kane et al., 2013). Importantly, we found that MX2 Δ1-25 is an effective suppressor of the IFN response (Figure 3) and that the endogenous MX2 protein is the target for this phenotype, with competition for CA binding being the underpinning mechanism driving this effect. Consistent with the G domain contributing to CA binding, the short isoform of MX2 is a stronger competitor against MX2 ($G_{MX1}$) than against wild-type MX2 (Figure 4B). These results point to the short isoform of MX2 playing a biological role during virus infection and invoke a scenario where IFN-stimulated cells can limit the activity of full-length MX2. Though the basis for the regulatory circuit is not yet known, we speculate that full-length MX2 exerts deleterious effects on cell function, such as impeded nuclear transport (Kane et al., 2018), that need to be minimized. In this regard, it is interesting that a number of ISGs express shorter protein isoforms that, at least in the case of mitochondrial antiviral-signaling (MAVS) protein, can exert a checkpoint-like function that dampens the activity of their full-length counterparts (Brubaker et al., 2014).

Structural data highlight the role played by the G domain in the oligomerization of both MX1 and MX2 (Gao et al., 2010, 2011; Alvarez et al., 2017). Therefore, it could be argued that different oligomerization kinetics between MX2 Δ1-25 and MX2 or MX2 ($G_{MX1}$) could be affecting the antiviral activity of the proteins. Nevertheless, in infection experiments carried out in the presence of the monomeric variant MX2 Δ1-25 M574D (Buffone et al., 2015; Dicks et al., 2015), where interactions between different MX2 species are prevented, wild-type MX2 still shows a more potent inhibition of HIV-1 than does MX2 ($G_{MX1}$) (Figure 4C). This finding is in good agreement with results obtained by overexpression of the same monomeric short isoform in the presence of IFNα, pointing to competition for CA binding as the mechanistic basis for downregulation of the full-length protein.

Detailed mapping of G-domain residues involved in capsid binding identified three amino acids as being important: glycine-184, asparagine-260, and glutamine-351 (Figure 5C). Infection experiments performed in the presence of competing MX2 Δ1-25 showed that the combined triple-mutant G184S/N260S/Q351E

has the weakest antiviral phenotype, with each single mutant having effects that scored as intermediate (Figure 5D). Interestingly, these three residues are located at distance from each other and are positioned within different surfaces of the G domain (Figure S7). The basis for the effects of these mutations on capsid interactions remains to be determined and may involve direct binding and/or conformational changes. Obtaining high-resolution structural information on the complex between MX2 and the HIV-1 capsid lattice will help answer such questions.

Our description of the MX2 G-domain-capsid interaction is concordant with the conclusions of a recent study showing that higher-order MX2 tubular structures expose their G domains toward the outer surface (Alvarez et al., 2017), possibly allowing interactions with incoming viral capsids. We can envisage a number of benefits for MX2 containing two CA-binding sites. First, improved capsid binding and enhanced antiviral efficacy may be particularly important during the initial phases of an IFN-induced antiviral response, as ISGs have yet to reach optimal expression levels. Second, the MX2 G-domain-capsid interaction allows fine-tuning of full-length MX2 activity by its short isoform, possibly limiting any deleterious effect that the long isoform may have on cellular functions. Third, MX2 interacts with other cellular factors, notably nucleoporins (Dicks et al., 2018; Kane et al., 2018), as part of the antiviral process; this interaction also requires the NTD of MX2 such that additional CA-binding sites within MX2 may facilitate simultaneous contact with different ligands, a property that is likely amplified by MX2 oligomerization. In sum, we propose that the interaction of the G domain of MX2 with the capsid not only improves HIV-1 recognition, but may also be required for the precise control of antiviral activity.

## STAR★METHODS

Detailed methods are provided in the online version of this paper and include the following:

- KEY RESOURCES TABLE
- LEAD CONTACT AND MATERIALS AVAILABILITY
- EXPERIMENTAL MODEL AND SUBJECT DETAILS
  - Cells
  - HIV-1 and lentiviral vector infections
- METHOD DETAILS
  - Protein expression and purification
  - *In vitro* assembly of CANC complexes
  - CANC pull-down
  - Plasmid constructs
- QUANTIFICATION AND STATISTICAL ANALYSIS
- DATA AND CODE AVAILABILITY

## SUPPLEMENTAL INFORMATION

Supplemental Information can be found online at https://doi.org/10.1016/j.celrep.2019.10.009.

## ACKNOWLEDGMENTS

We thank Darja Pollpeter, Caroline Goujon, and Stelios Papaioannou for the provision of reagents and helpful discussions. The work was supported by the U.K. Medical Research Council (G1000196); the Wellcome Trust (106223/Z/14/Z); the National Institute of General Medical Sciences (U54 GM103368); and the Department of Health via a NIH Research comprehensive Biomedical Research Centre award to Guy's and St. Thomas' NHS Foundation Trust in partnership with King's College London and King's College Hospital NHS Foundation Trust. J.M.J.-G. is a long-term fellow of the European Molecular Biology Organization (EMBO) (ALTF 663-2016).

## AUTHOR CONTRIBUTIONS

G.B. and M.H.M. designed the study and wrote the manuscript with input from all co-authors; G.B. carried out the experiments and analyzed the data, with contributions from M.D.J.D. in Figure 6, J.M.J.-G. in Figure 5, and N.H.A. in Figure 6 and appendix Figure 4; M.D.J.D., J.M.J.-G., and L.A. contributed to the execution of experiments and provided reagents; and M.H.M. supervised all aspects of the project. The ORCID number for G.B. is 0000-0003-0548-7690. The ORCID number for M.D.J.D. is 0000-0003-1909-7095. The ORCID number for J.M.J.-G. is 0000-0002-1726-8033. The ORCID number for L.A. is 0000-0002-9451-6117. The ORCID number for M.H.M. is 0000-0002-7699-2064.

## DECLARATION OF INTERESTS

The authors declare no competing interests.

Received: April 1, 2019
Revised: July 18, 2019
Accepted: October 2, 2019
Published: November 12, 2019

## REFERENCES

Adachi, A., Gendelman, H.E., Koenig, S., Folks, T., Willey, R., Rabson, A., and Martin, M.A. (1986). Production of acquired immunodeficiency syndrome-associated retrovirus in human and nonhuman cells transfected with an infectious molecular clone. J. Virol. *59*, 284–291.

Alvarez, F.J.D., He, S., Perilla, J.R., Jang, S., Schulten, K., Engelman, A.N., Scheres, S.H.W., and Zhang, P. (2017). CryoEM structure of MxB reveals a novel oligomerization interface critical for HIV restriction. Sci. Adv. *3*, e1701264.

Bharat, T.A., Davey, N.E., Ulbrich, P., Riches, J.D., de Marco, A., Rumlova, M., Sachse, C., Ruml, T., and Briggs, J.A. (2012). Structure of the immature retroviral capsid at 8 Å resolution by cryo-electron microscopy. Nature *487*, 385–389.

Black, L.R., and Aiken, C. (2010). TRIM5alpha disrupts the structure of assembled HIV-1 capsid complexes in vitro. J. Virol. *84*, 6564–6569.

Bourke, N.M., Napoletano, S., Bannan, C., Ahmed, S., Bergin, C., McKnight, Á., and Stevenson, N.J. (2018). Control of HIV infection by IFN-$\alpha$: implications for latency and a cure. Cell. Mol. Life Sci. *75*, 775–783.

Brubaker, S.W., Gauthier, A.E., Mills, E.W., Ingolia, N.T., and Kagan, J.C. (2014). A bicistronic MAVS transcript highlights a class of truncated variants in antiviral immunity. Cell *156*, 800–811.

Buffone, C., Schulte, B., Opp, S., and Diaz-Griffero, F. (2015). Contribution of MxB oligomerization to HIV-1 capsid binding and restriction. J. Virol. *89*, 3285–3294.

Bulli, L., Apolonia, L., Kutzner, J., Pollpeter, D., Goujon, C., Herold, N., Schwarz, S.M., Giernat, Y., Keppler, O.T., Malim, M.H., and Schaller, T. (2016). Complex Interplay between HIV-1 Capsid and MX2-Independent Alpha Interferon-Induced Antiviral Factors. J. Virol. *90*, 7469–7480.

Busnadiego, I., Kane, M., Rihn, S.J., Preugschas, H.F., Hughes, J., Blanco-Melo, D., Strouvelle, V.P., Zang, T.M., Willett, B.J., Boutell, C., et al. (2014). Host and viral determinants of Mx2 antiretroviral activity. J. Virol. *88*, 7738–7752.

Campbell, S., and Vogt, V.M. (1995). Self-assembly in vitro of purified CA-NC proteins from Rous sarcoma virus and human immunodeficiency virus type 1. J. Virol. *69*, 6487–6497.

Crameri, M., Bauer, M., Caduff, N., Walker, R., Steiner, F., Franzoso, F.D., Gujer, C., Boucke, K., Kucera, T., Zbinden, A., et al. (2018). MxB is an interferon-induced restriction factor of human herpesviruses. Nat. Commun. *9*, 1980.

Dharan, A., Opp, S., Abdel-Rahim, O., Keceli, S.K., Imam, S., Diaz-Griffero, F., and Campbell, E.M. (2017). Bicaudal D2 facilitates the cytoplasmic trafficking and nuclear import of HIV-1 genomes during infection. Proc. Natl. Acad. Sci. USA *114*, E10707–E10716.

Dick, R.A., Zadrozny, K.K., Xu, C., Schur, F.K.M., Lyddon, T.D., Ricana, C.L., Wagner, J.M., Perilla, J.R., Ganser-Pornillos, B.K., Johnson, M.C., et al. (2018). Inositol phosphates are assembly co-factors for HIV-1. Nature *560*, 509–512.

Dicks, M.D., Goujon, C., Pollpeter, D., Betancor, G., Apolonia, L., Bergeron, J.R., and Malim, M.H. (2015). Oligomerization Requirements for MX2-Mediated Suppression of HIV-1 Infection. J. Virol. *90*, 22–32.

Dicks, M.D.J., Betancor, G., Jimenez-Guardeño, J.M., Pessel-Vivares, L., Apolonia, L., Goujon, C., and Malim, M.H. (2018). Multiple components of the nuclear pore complex interact with the amino-terminus of MX2 to facilitate HIV-1 restriction. PLoS Pathog. *14*, e1007408.

Doyle, T., Goujon, C., and Malim, M.H. (2015). HIV-1 and interferons: who's interfering with whom? Nat. Rev. Microbiol. *13*, 403–413.

Fouchier, R.A., Meyer, B.E., Simon, J.H., Fischer, U., and Malim, M.H. (1997). HIV-1 infection of non-dividing cells: evidence that the amino-terminal basic region of the viral matrix protein is important for Gag processing but not for post-entry nuclear import. EMBO J. *16*, 4531–4539.

Frese, M., Kochs, G., Feldmann, H., Hertkorn, C., and Haller, O. (1996). Inhibition of bunyaviruses, phleboviruses, and hantaviruses by human MxA protein. J. Virol. *70*, 915–923.

Fribourgh, J.L., Nguyen, H.C., Matreyek, K.A., Alvarez, F.J.D., Summers, B.J., Dewdney, T.G., Aiken, C., Zhang, P., Engelman, A., and Xiong, Y. (2014). Structural insight into HIV-1 restriction by MxB. Cell Host Microbe *16*, 627–638.

Fricke, T., White, T.E., Schulte, B., de Souza Aranha Vieira, D.A., Dharan, A., Campbell, E.M., Brandariz-Nuñez, A., and Diaz-Griffero, F. (2014). MxB binds to the HIV-1 core and prevents the uncoating process of HIV-1. Retrovirology *11*, 68.

Gao, S., von der Malsburg, A., Paeschke, S., Behlke, J., Haller, O., Kochs, G., and Daumke, O. (2010). Structural basis of oligomerization in the stalk region of dynamin-like MxA. Nature *465*, 502–506.

Gao, S., von der Malsburg, A., Dick, A., Faelber, K., Schröder, G.F., Haller, O., Kochs, G., and Daumke, O. (2011). Structure of myxovirus resistance protein a reveals intra- and intermolecular domain interactions required for the antiviral function. Immunity *35*, 514–525.

Gordien, E., Rosmorduc, O., Peltekian, C., Garreau, F., Bréchot, C., and Kremsdorf, D. (2001). Inhibition of hepatitis B virus replication by the interferon-inducible MxA protein. J. Virol. *75*, 2684–2691.

Goujon, C., Moncorgé, O., Bauby, H., Doyle, T., Ward, C.C., Schaller, T., Hué, S., Barclay, W.S., Schulz, R., and Malim, M.H. (2013). Human MX2 is an interferon-induced post-entry inhibitor of HIV-1 infection. Nature *502*, 559–562.

Goujon, C., Moncorgé, O., Bauby, H., Doyle, T., Barclay, W.S., and Malim, M.H. (2014). Transfer of the amino-terminal nuclear envelope targeting domain of human MX2 converts MX1 into an HIV-1 resistance factor. J. Virol. *88*, 9017–9026.

Goujon, C., Greenbury, R.A., Papaioannou, S., Doyle, T., and Malim, M.H. (2015). A triple-arginine motif in the amino-terminal domain and oligomerization are required for HIV-1 inhibition by human MX2. J. Virol. *89*, 4676–4680.

Gross, I., Hohenberg, H., and Kräusslich, H.G. (1997). In vitro assembly properties of purified bacterially expressed capsid proteins of human immunodeficiency virus. Eur. J. Biochem. *249*, 592–600.

Hwang, S.S., Boyle, T.J., Lyerly, H.K., and Cullen, B.R. (1991). Identification of the envelope V3 loop as the primary determinant of cell tropism in HIV-1. Science *253*, 71–74.

Kane, M., Yadav, S.S., Bitzegeio, J., Kutluay, S.B., Zang, T., Wilson, S.J., Schoggins, J.W., Rice, C.M., Yamashita, M., Hatziioannou, T., and Bieniasz, P.D. (2013). MX2 is an interferon-induced inhibitor of HIV-1 infection. Nature *502*, 563–566.

Kane, M., Rebensburg, S.V., Takata, M.A., Zang, T.M., Yamashita, M., Kvaratskhelia, M., and Bieniasz, P.D. (2018). Nuclear pore heterogeneity influences HIV-1 infection and the antiviral activity of MX2. eLife *7*, e35738.

Kochs, G., and Haller, O. (1999). Interferon-induced human MxA GTPase blocks nuclear import of Thogoto virus nucleocapsids. Proc. Natl. Acad. Sci. USA *96*, 2082–2086.

Li, X., and Sodroski, J. (2008). The TRIM5alpha B-box 2 domain promotes cooperative binding to the retroviral capsid by mediating higher-order self-association. J. Virol. *82*, 11495–11502.

Liu, Z., Pan, Q., Ding, S., Qian, J., Xu, F., Zhou, J., Cen, S., Guo, F., and Liang, C. (2013). The interferon-inducible MxB protein inhibits HIV-1 infection. Cell Host Microbe *14*, 398–410.

Matreyek, K.A., Wang, W., Serrao, E., Singh, P.K., Levin, H.L., and Engelman, A. (2014). Host and viral determinants for MxB restriction of HIV-1 infection. Retrovirology *11*, 90.

Mitchell, P.S., Patzina, C., Emerman, M., Haller, O., Malik, H.S., and Kochs, G. (2012). Evolution-guided identification of antiviral specificity determinants in the broadly acting interferon-induced innate immunity factor MxA. Cell Host Microbe *12*, 598–604.

Pavlovic, J., Zürcher, T., Haller, O., and Staeheli, P. (1990). Resistance to influenza virus and vesicular stomatitis virus conferred by expression of human MxA protein. J. Virol. *64*, 3370–3375.

Pitossi, F., Blank, A., Schröder, A., Schwarz, A., Hüssi, P., Schwemmle, M., Pavlovic, J., and Staeheli, P. (1993). A functional GTP-binding motif is necessary for antiviral activity of Mx proteins. J. Virol. *67*, 6726–6732.

Ponten, A., Sick, C., Weeber, M., Haller, O., and Kochs, G. (1997). Dominant-negative mutants of human MxA protein: domains in the carboxy-terminal moiety are important for oligomerization and antiviral activity. J. Virol. *71*, 2591–2599.

Price, A.J., Fletcher, A.J., Schaller, T., Elliott, T., Lee, K., KewalRamani, V.N., Chin, J.W., Towers, G.J., and James, L.C. (2012). CPSF6 defines a conserved capsid interface that modulates HIV-1 replication. PLoS Pathog. *8*, e1002896.

Schaller, T., Bulli, L., Pollpeter, D., Betancor, G., Kutzner, J., Apolonia, L., Herold, N., Burk, R., and Malim, M.H. (2017). Effects of Inner Nuclear Membrane Proteins SUN1/UNC-84A and SUN2/UNC-84B on the Early Steps of HIV-1 Infection. J. Virol. *91*, e00463-17.

Schilling, M., Bulli, L., Weigang, S., Graf, L., Naumann, S., Patzina, C., Wagner, V., Bauersfeld, L., Goujon, C., Hengel, H., et al. (2018). Human MxB protein is a pan-herpesvirus restriction factor. J. Virol. *92*, e01056-18.

Schneider, C.A., Rasband, W.S., and Elicieri, K.W. (2012). NIH Image to ImageJ: 25 years of image analysis. Nat Methods. *9*, 671–675.

Schulte, B., Buffone, C., Opp, S., Di Nunzio, F., De Souza Aranha Vieira, D.A., Brandariz-Nuñez, A., and Diaz-Griffero, F. (2015). Restriction of HIV-1 Requires the N-Terminal Region of MxB as a Capsid-Binding Motif but Not as a Nuclear Localization Signal. J. Virol. *89*, 8599–8610.

Smaga, S.S., Xu, C., Summers, B.J., Digianantonio, K.M., Perilla, J.R., and Xiong, Y. (2018). MxB restricts HIV-1 by targeting the tri-hexamer interface of the viral capsid. bioRxiv.org. https://doi.org/10.1016/j.str.2019.04.015.

Staeheli, P., and Haller, O. (2018). Human MX2/MxB: a Potent Interferon-Induced Postentry Inhibitor of Herpesviruses and HIV-1. J. Virol. *92*, e00709-18.

Tan, S. (2001). A modular polycistronic expression system for overexpressing protein complexes in Escherichia coli. Protein Expr. Purif. *21*, 224–234.

Valle-Casuso, J.C., Di Nunzio, F., Yang, Y., Reszka, N., Lienlaf, M., Arhel, N., Perez, P., Brass, A.L., and Diaz-Griffero, F. (2012). TNPO3 is required for HIV-1 replication after nuclear import but prior to integration and binds the HIV-1 core. J. Virol. *86*, 5931–5936.

Verhelst, J., Hulpiau, P., and Saelens, X. (2013). Mx proteins: antiviral gatekeepers that restrain the uninvited. Microbiol. Mol. Biol. Rev. *77*, 551–566.

Verhelst, J., Spitaels, J., Nürnberger, C., De Vlieger, D., Ysenbaert, T., Staeheli, P., Fiers, W., and Saelens, X. (2015). Functional Comparison of Mx1 from Two Different Mouse Species Reveals the Involvement of Loop L4 in the Antiviral Activity against Influenza A Viruses. J. Virol. *89*, 10879–10890.

# STAR★METHODS

## KEY RESOURCES TABLE

| REAGENT or RESOURCE | SOURCE | IDENTIFIER |
|---|---|---|
| Antibodies | | |
| Goat polyclonal anti-MX2 N17 | Santa Cruz Biotechnologies | Cat#sc-47197; RRID:AB_2147726 |
| Mouse anti-goat-HRP (horse radish peroxidase) | Santa Cruz Biotechnologies | Cat#sc-2354; RRID:AB_628490 |
| Mouse monoclonal anti-Flag-HRP M2 | Sigma | Cat#F3165-1MG; RRID:AB_259529 |
| Rat monoclonal anti-HA-HRP 3F10 | Sigma | Cat#12 013 819 001; RRID:AB_390917 |
| Mouse monoclonal anti-CA 24.2 | Fouchier et al., 1997 | N/A |
| Mouse monoclonal anti-tubulin | Sigma | Cat#T5168-.2ML; RRID:AB_477579 |
| Goat monoclonal anti-mouse IRDye fluorescent | Li-Cor Biosciences | Cat#926-32210; RRID:AB_621842 |
| Rabbit polyclonal anti-MX1 | Abcam | Cat#ab95926; RRID:AB_10677452 |
| Bacterial and Virus Strains | | |
| HIV-$1_{IIIB}$ | Hwang et al., 1991 | N/A |
| HIV-$1_{NL4.3}$ | Adachi et al., 1986 | N/A |
| *Escherichia coli* Rosetta (DE3) | Merck Millipore | Cat#70954 |
| Chemicals, Peptides, and Recombinant Proteins | | |
| Isopropyl β-D-1-thiogalactopyranoside (IPTG) | ThermoFisher Scientific | Cat#R0393 |
| Ammonium sulfate | Sigma | Cat#A4418-1KG |
| Poly(ethyleneimine) solution | Fluka Analytical | Cat#3880-100ML |
| MES hydrate | Sigma | Cat#M8250-250G |
| Dithiothreitol (DTT) | ThermoFisher Scientific | Cat#20290 |
| Sodium chloride | VWR Chemicals | Cat#27810.364 |
| Phenylmethanesulfonyl fluoride | Sigma | Cat#P7626-1G |
| Tris Base | Fisher Scientific | Cat#BP152-5 |
| Potassium chloride | Sigma | Cat#P9541-1KG |
| Sucrose | Sigma | Cat#S0398-1KG |
| HiTrap SP HP columns | Ge Healthcare Life Sciences | Cat#17115201 |
| cOmplete Mini protease inhibitor cocktail tablets | Sigma | Cat#11836153001 |
| Glycerol | Sigma | Cat#G5516-1L |
| DMEM, high glucose, GlutaMAX Supplement | ThermoFisher Scientific | Cat# 10566016 |
| Fetal Bovine Serum | ThermoFisher Scientific | Cat#10270 |
| Penicillin Streptomycin (Pen Strep) | ThermoFisher Scientific | Cat#15140-122 |
| EcoRI-HF | New England Biolabs | Cat#R3101S |
| NotI-HF | New England Biolabs | Cat#R3189S |
| XhoI | New England Biolabs | Cat#R0146L |
| BamHI | New England Biolabs | Cat#R)136M |
| Puromycin | ThermoFisher Scientific | Cat#A1113802 |
| IFNα-2b | INTRON A, Merck, Sharpe & Dohme Corp. | N/A |
| Doxycycline | Sigma | Cat#D9891 |
| EDTA | Invitrogen | Cat#AM92260G |
| Critical Commercial Assays | | |
| HIV-1 p24 antigen ELISA kit | PerkinElmer | Cat#NEK050001KT |
| Tropix Galacto-Star kit | ThermoFisher Scientific | Cat#T1014 |
| Deposited Data | | |
| PDB file | Fribourgh et al., 2014 | 4WHJ |

(*Continued on next page*)

| *Continued* | | |
|---|---|---|
| REAGENT or RESOURCE | SOURCE | IDENTIFIER |
| Experimental Models: Cell Lines | | |
| HeLa-TZMbl | NIH AIDS Reagent Program | Cat#8129 |
| 293T | ATCC | Cat#CRL-3216 |
| U87-MG CD4/CXCR4 | Goujon et al., 2013 | N/A |
| Oligonucleotides | | |
| TG50: TGTGTGTGTGTGTGTGTGTGTG TGTGTGTGTGTGTGTGTGTGTGTGTGTG | This paper | N/A |
| Recombinant DNA | | |
| pST39 | Tan, 2001 | N/A |
| pCAGGs | Goujon et al., 2014 | N/A |
| EasiLV-MCS | Goujon et al., 2013 | N/A |
| PuromycinR | Goujon et al., 2013 | N/A |
| Software and Algorithms | | |
| ImageJ | Schneider et al., 2012 | https://imagej.nih.gov/ij/ |
| GraphPad Prism version 6.0.0 | GraphPad Software | https://www.graphpad.com |

## LEAD CONTACT AND MATERIALS AVAILABILITY

For further information and requests for reagents, please contact the Lead Contact, Michael H. Malim (michael.malim@kcl.ac.uk). The plasmids generated in this study are available upon request without restriction.

## EXPERIMENTAL MODEL AND SUBJECT DETAILS

### Cells

Control CRISPR U87-MG cells and MX2 CRISPR U87-MG cells have been described before (Dicks et al., 2018). The CRISPR modified cells, U87-MG CD4/CXCR4 cells (Goujon et al., 2013), HeLa-TZMbl indicator cells (NIH AIDS Reagent Program) and 293T cells were cultured in Dulbecco's modified Eagle medium (DMEM) supplemented with heat-inactivated fetal bovine serum (10%), L-glutamine, penicillin (100 U/ml) and streptomycin (100 μg/ml).

### HIV-1 and lentiviral vector infections

Wild-type HIV-$1_{NL4-3}$ (Adachi et al., 1986) stocks were obtained by filtration of culture medium from transfected 293T cells, and $p24^{Gag}$ content determined by ELISA according to the manufacturer's instructions (Perkin-Elmer). U87-MG CD4/CXCR4 cells were transduced with either puromycinR encoding Flag-tagged MX2, MX1, MX2 ($G_{MX1}$), MX2 G184S, MX2 N260S, MX2 Q351E or MX2 G184S/N260S/Q351E alone, or together with EasiLV encoding HA-tagged MX2 Δ1-25 or MX2 Δ1-25 M574D. After selection for puromycin resistant cells for 48 h, $2.5x10^4$ cells were seeded in a 24 well plate, challenged with HIV-$1_{NL4-3}$ (corresponding to 185 ng $p24^{Gag}$ for experiments comparing MX1, MX2 and MX2 ($G_{MX1}$); or 350 ng $p24^{Gag}$ for experiments comparing wild-type MX2 and mutants) and the medium replaced after 6 h. For every subsequent 24 h period, for a total of 120 h, medium was filtered and stored at $-80°$C. To quantify released infectious virus at each time point, HeLa-TZMbl indicator cells (NIH AIDS Reagent Program) were challenged and productive infection determined at 48 h by measuring chemiluminescent β-galactosidase activity using the Tropix Galacto-Star system according to the manufacturer's instructions (Applied Biosystems).

HIV-1 based lentiviral particles expressing GFP (HIV-1/GFP) were produced as described previously (Goujon et al., 2013). Infectivity experiments using EasiLV and/or puromycinR transduced U87-MG CD4/CXCR4 cultures were carried out as previously described, with the percent of cells expressing GFP enumerated by flow cytometry (FACSCanto II; BD Biosciences) at 48 h post infection (Dicks et al., 2015). For experiments where U87-MG CD4/CXCR4 cells were treated with IFNα, cells were transduced with EasiLV vectors expressing either luciferase, MX2, MX2 Δ1-25 or MX2 Δ1-25 M574D and the medium replaced 6 h later for fresh DMEM containing 0.5 μg/ml doxycycline. 24 h later, 500 U/ml of IFNα-2b (INTRON A, Merck, Sharpe & Dohme Corp.) was added, and after a further 24 h the cells were challenged with HIV-1/GFP. After 48 h, productive infection was monitored by flow cytometry.

## METHOD DETAILS

### Protein expression and purification

DNA encoding HIV-$1_{IIIB}$ (Hwang et al., 1991) Capsid-Nucleocapsid (CANC, corresponding to Gag residues 133-432) was PCR amplified and inserted into pST39 (Tan, 2001) using the NdeI and BamHI sites. Protein was expressed in *Escherichia coli* Rosetta (DE3)

cells (Merck Millipore) as previously described (Schaller et al., 2017). Briefly, CANC expression was induced with 1 mM of isopropyl β-D-thiogalactopyranoside (IPTG) for 6 h at 30°C. Cells were collected by centrifugation and lysed by sonication. Cell debris were removed by centrifugation at 30000 g for 20 min. Nucleic acids present in the supernatant were removed by adding of 0.11 volumes of 2 M ammonium sulfate and the same volume of 10% polyethylenimine (PEI) pH 8.0. CANC was precipitated from the supernatant by adding 0.35 volumes of saturated ammonium sulfate and centrifugation at 10000 g for 15 min. Pelleted protein was resuspended in 50 mM MES pH 6.5, 1 mM EDTA, 1 mM DTT, 0.5 M NaCl, 10% glycerol, 1 mM PMSF and then diluted with the same buffer lacking NaCl to a final concentration of 0.2 M NaCl. This was cleared by centrifugation and CANC polished by cation-exchange chromatography on a 5 mL HiTrap SP HP column (GE Healthcare) using 50 mM MES pH 6.5, 1 mM EDTA, 1 mM DTT, 0.2 M NaCl, 10% glycerol, 1 mM PMSF as the equilibration buffer. CANC was eluted following a linear gradient resulting from mixing the equilibration buffer with the same buffer adjusted to 1 M NaCl. Protein-enriched fractions were pooled and CANC was precipitated by adding one volume of saturated ammonium sulfate. Finally, resulting CANC was resuspended at 200 μM in 30 mM MES pH 6, 1 mM EDTA, 0.5 M NaCl, 10 mM DTT, and stored at −80°C.

### *In vitro* assembly of CANC complexes

Assembled CANC complexes were obtained by diluting the purified protein to 40 μM in 50 mM Tris-HCl pH 8, 100 mM NaCl with 5 μM TG50 oligonucleotide and overnight incubation at room temperature. The sequence of TG50 is: 5′-25(TG)-3′

### CANC pull-down

293T cells transfected with vectors expressing hemagglutinin (HA)- or Flag-tagged proteins were harvested and resuspended in hypotonic lysis buffer (10 mM Tris-HCl pH 8, 10 mM KCl, 1x protease inhibitor cocktail [Roche]) and lysed using a Dounce homogenizer. Lysates were cleared by centrifugation at 20000 g at 4°C for 15 min. In pull-down experiments, 200 μL of cell lysates were mixed with 40 μL of 40 μM assembled CANC (an input sample was reserved from this mix) or 40 μL of Capsid assembly buffer (Cab, 50 mM Tris-HCl pH 8, 100 mM NaCl) containing 5 μM TG50 and incubated under gently agitation at room temperature for 1 h. In the case of competition experiments, 200 μl of cell lysate expressing HA-tagged proteins and 200 μl of cell lysate expressing Flag-tagged proteins were mixed with 80 μL of 40 μM assembled CANC. The mixture was then overlaid onto a 250 μL sucrose cushion (70% weight/volume) and centrifuged at 20000 g at room temperature for 10 min. A sample of the supernatant was withdrawn for further analysis. The pellet was washed with 500 μL wash buffer (50 mM Tris-HCl pH 8, 50 mM NaCl, 5 mM KCl), and re-centrifuged at 10000 g at room temperature for 8 min. Finally, the pellet was resuspended in 50 μL of 1x SDS-PAGE loading buffer. Input, supernatant and pellet fractions were analyzed by immunoblot using anti-MX2 goat polyclonal antibody (N17, Santa Cruz Biotechnology) and mouse anti-goat horseradish peroxidase (HRP)-conjugated monoclonal antibody (Santa Cruz), anti-Flag HRP-conjugated mouse monoclonal M2 (Sigma), anti-HA HRP-conjugated rat monoclonal antibody (Sigma), anti-CA mouse monoclonal 24.2 (Fouchier et al., 1997) or anti-tubulin mouse monoclonal (Sigma) together with goat anti-mouse IRDye fluorescent monoclonal antibody (Li-Cor Biosciences) and detected using a LI-COR Odyssey FC imaging system (LI-COR Biosciences).

### Plasmid constructs

pCAGGs plasmid (Adgene) was used for the expression of cDNA fragments used in transfection experiments, after PCR amplification and inclusion of either Flag- or HA-tags, using EcoRI-XhoI or NotI-XhoI sites. Fragments encoding the chimeric proteins GFP ($NTD_{MX2}$), GFP ($G_{MX2}$), MX2 ΔNTD ($92\text{-}234_{MX1}$), MX2 ΔNTD ($235\text{-}322_{MX1}$) or MX2 ΔNTD ($323\text{-}387_{MX1}$) were produced by standard overlapping PCR. Truncated fragments encoding MX2 ΔNTD, MX1 ($NTD_{MX2}$) or GFP ($NTD_{MX2}$) were obtained from full-length versions (Goujon et al., 2014) using PCR. Site-directed mutagenesis was used to obtain single or multiply mutated constructs, using PCR amplification methods. Where required, DNA constructs were cloned into the doxycycline-inducible lentiviral vector EasiLV-MCS (Goujon et al., 2013) or into a lentiviral vector expressing a CD4-IRES-puromycin N-acetyltransferase expression cassette (puromycinR) (Goujon et al., 2013) using BamHI-XhoI restriction sites.

## QUANTIFICATION AND STATISTICAL ANALYSIS

All statistical analyses were performed using GraphPad Prism 6.0 (GraphPad Software Inc.). For each experiment, mean ± SD was calculated and the unpaired t test was applied in all cases, considering $p < 0.05$ as the level of statistical significance. Corresponding results from statistical analyses performed can be found in the relevant figure legends.

## DATA AND CODE AVAILABILITY

This study did not generate any unique datasets or code.

**Cell Reports, Volume *29***

## Supplemental Information

# The GTPase Domain of MX2 Interacts with the HIV-1 Capsid, Enabling Its Short Isoform to Moderate Antiviral Restriction

**Gilberto Betancor, Matthew D.J. Dicks, Jose M. Jimenez-Guardeño, Nabil H. Ali, Luis Apolonia, and Michael H. Malim**

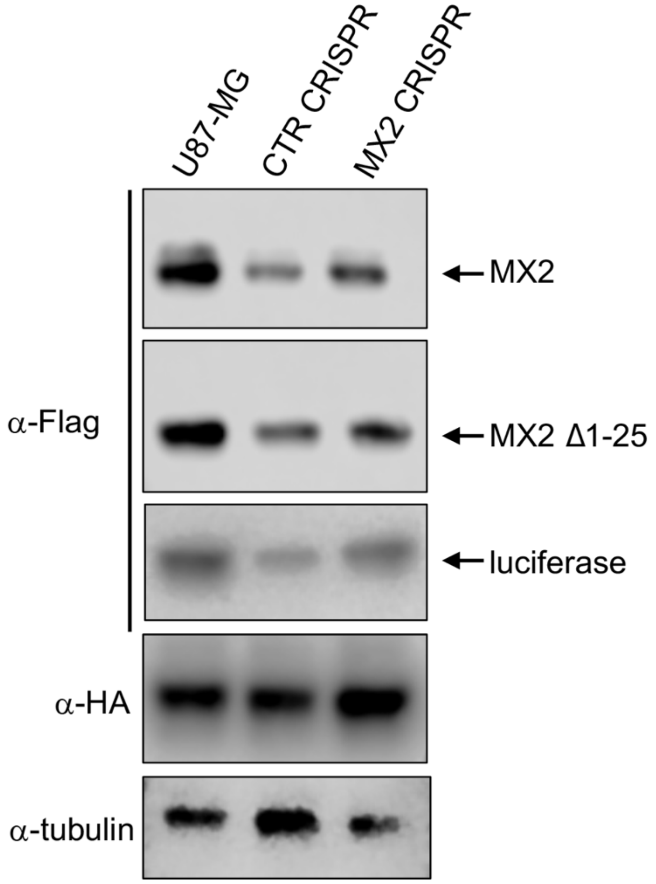


**Figure S1. Protein levels in U87-MG parental cells and CTR CRISPR and MX2 CRISPR derived cell lines** (related to Figure 3A-C).

Protein expression in cells transduced with Flag-tagged MX2 (resistant to CRISPR guide), MX2 Δ1-25, or Luc or HA-tagged MX2 Δ1-25 M574D was analyzed by immunoblot, with α-tubulin included as the loading control.

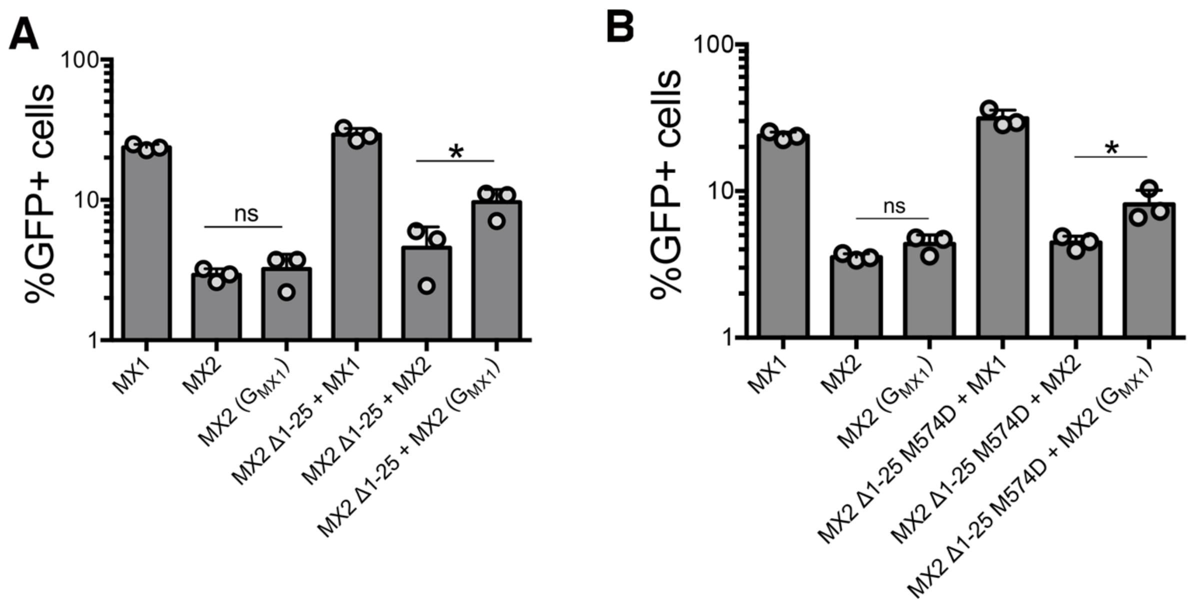


**Figure S2. Wild type MX2 inhibits HIV-1 more potently than MX2 ($G_{MX1}$)** (related to Figure 4B and C).

A U87-MG CD4/CXCR4 cells were transduced with EasiLV expressing MX2 Δ1-25 together with either MX1-, MX2- or MX2 ($G_{MX1}$)-expressing lentiviral vectors carrying puromycinR. Two days later, transduced cells were selected with 1 µg/ml puromycin for 48 h and induced with doxycycline. Cells were then infected with an HIV-1-based lentiviral vector expressing GFP, and the percentage of GFP-expressing cells was evaluated by flow cytometry at 48 h (n = 3, mean ± SD; *$p$-value < 0.05; (ns) non-significant; unpaired t-test).

B Same as A, but expressing monomeric mutant MX2 Δ1-25 M574D instead of MX2 Δ1-25 (n = 3, mean ± SD; *$p$-value < 0.05; (ns) non-significant; unpaired t-test).

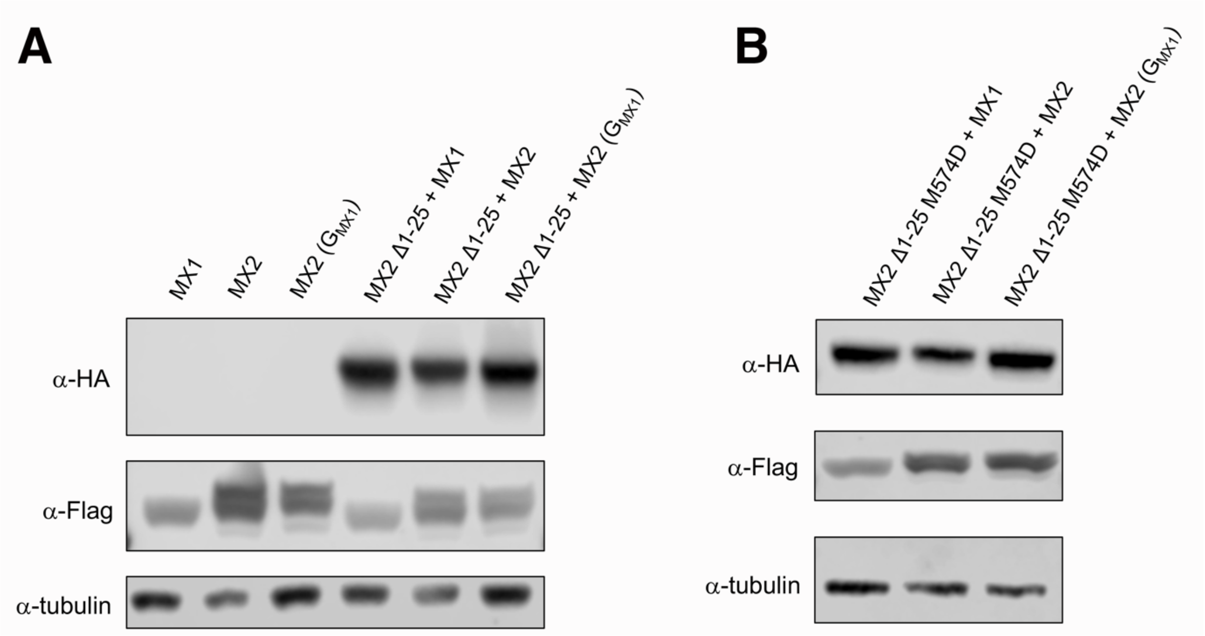


**Figure S3. Protein levels of MX1, MX2, MX2 ($G_{MX1}$) and MX2 Δ1-25 in viral replication experiments** (related to Figure 4B and C).

A Protein expression in cells transduced individually with Flag-tagged MX1, MX2 or MX2 ($G_{MX1}$), or together with HA-tagged MX2 Δ1-25 was analyzed by immunoblot, with α-tubulin included as the loading control.

B Protein expression in cells doubly transduced with Flag-tagged MX1, MX2 or MX2 ($G_{MX1}$), and HA-tagged MX2 Δ1-25 M574D was analyzed by immunoblot, with α-tubulin included as the loading control.

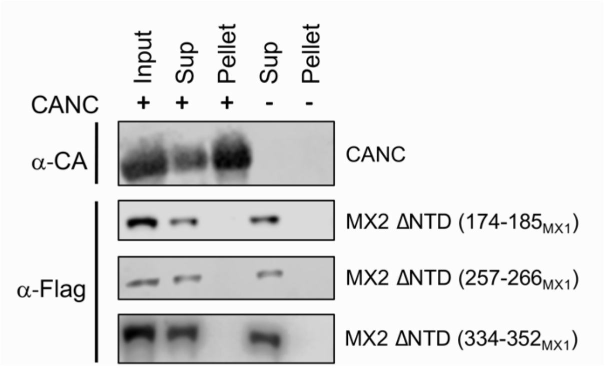


**Figure S4. Identification of regions of the MX2 G domain important for CANC binding** (related to Figure 5B).

MX2 Δ1-91 was mutated in positions 174-185, 257-266 or 334-352 by swapping MX2 residues for the corresponding residues of MX1. Lysates from expressing 293T cells were mixed with CANC assemblies, pelleted through a sucrose cushion, and Input, Sup and Pellet fractions were analyzed by immunoblot (n = at least 3). A representative immunoblot for CANC (α-CA) is shown.

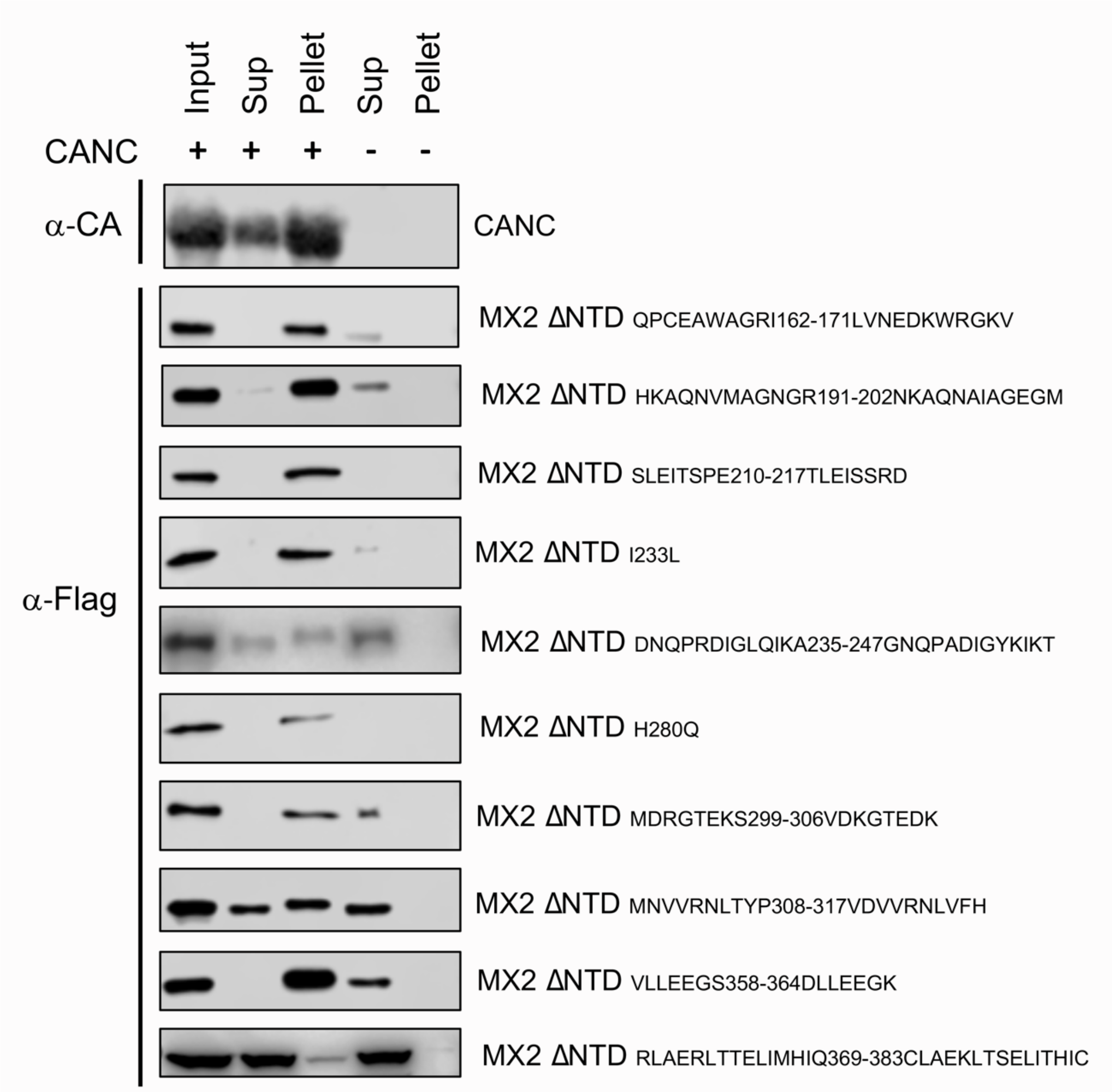


**Figure S5. Characterization of regions in the MX2 G domain involved in the interaction with the viral capsid** (related to Figure 5B).

Regions within the G domain of MX2 were swapped for the corresponding regions from MX1 in the MX2 ΔNTD background and tested for their ability to bind to assembled CANC complexes. Samples were pelleted through a sucrose cushion, and Input, Sup and Pellet fractions were analyzed by immunoblot (n = at least 3). A representative immunoblot for CANC (α-CA) is shown.

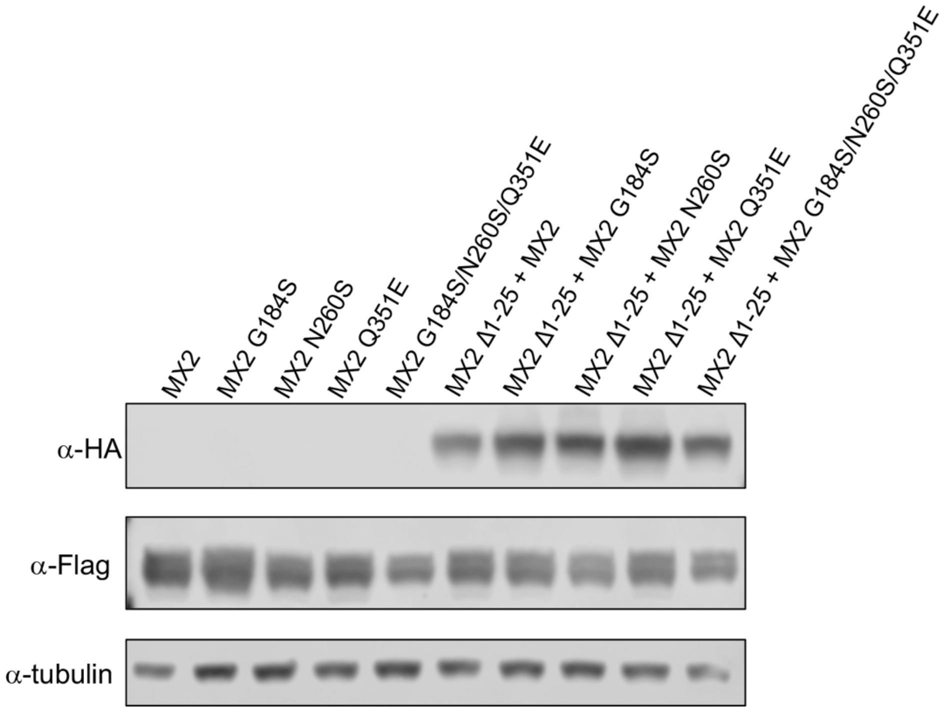


**Figure S6. Protein levels of wild type MX2, mutants G184S, N260S, Q351E, G184S/N260S/Q351E and MX2 Δ1-25 in viral replication experiments** (related to Figure 5D).

Protein expression in cells individually transduced with Flag-tagged MX2, MX2 G184S, MX2 N260S, MX2 Q351E or MX2 G184S/N260S/Q351E, or together with HA-tagged MX2 Δ1-25 was analyzed by immunoblot, with α-tubulin included as the loading control.

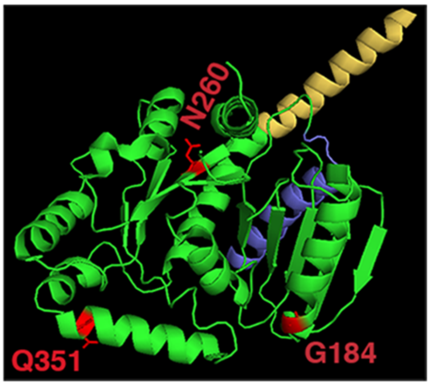


**Figure S7. Structure of the G domain of MX2** (related to Figure 5C)

Crystal structure of the G domain of MX2 showing bundle signaling elements (BSE) 1 and 2, presented in ribbon diagrams. The G domain is shown in green, BSE 1 in blue and BSE 2 in red. The side chains of the three key residues identified as relevant for CANC binding are shown in red. The structure was drawn with the PyMOL molecular viewer (http://www.pymol.org). Atom coordinates were taken from PDB file 4WHJ (Fribough et al., 2014).
